# Healable Glassy Metallosupramolecular Polymers

**DOI:** 10.1021/acsmacrolett.5c00317

**Published:** 2025-06-30

**Authors:** Chaninya Mak-iad, Luca Bertossi, Georges J. M. Formon, Christoph Weder

**Affiliations:** † 453785 Adolphe Merkle Institute, University of Fribourg, Chemin des Verdiers 4, 1700 Fribourg, Switzerland; ‡ NCCR Bio-inspired Materials, 27211 University of Fribourg, Chemin des Verdiers 4, 1700 Fribourg, Switzerland

## Abstract

Metallosupramolecular polymers (MSPs), formed through ligand–metal coordination, feature dynamic bonds that enable damage repair *via* reversible dissociation. Here, we report the synthesis of healable MSPs by modifying commercially available glycol-modified polyethylene terephthalate (PETG), an amorphous commodity polyester known for its durability, chemical resistance, and formability. The new materials were accessed through the Zn-catalyzed transesterification of PETG with ethylene glycol, end-functionalization of the resulting homobifunctional oligomers with the 2,6-bis­(1′-methylbenzimidazolyl)­pyridine (Mebip) ligand, and subsequent assembly of these macromonomers into MSPs *via* Zn^2+^ complexation. One of the MSPs made offers an attractive combination of high tensile strength (31 MPa), high Young’s Modulus (1 GPa), and excellent healability (94% in 2.5 min).

## Graphical abstract

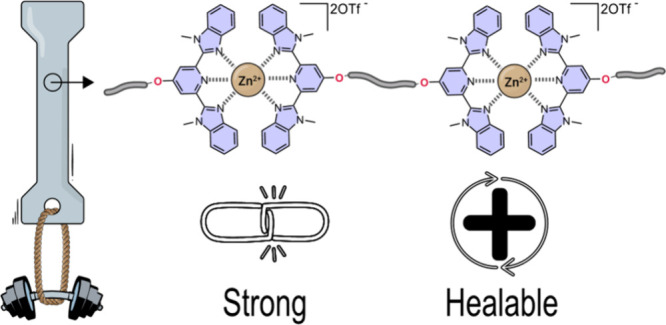

Healable materials mimic an essential function of natural materials, i.e., the ability to restore their structural and functional integrity after being damaged,^1,2^ either autonomously or through stimulus-induced repair.^3^ While all thermoplastic polymers heal when heated above their glass transition or melting temperature, this process is generally slow, due to the inverse relationship between the polymers’ chain length and their diffusion and re-entanglement rates.^4,5^ Moreover, maintaining the shape of an object under these conditions is challenging. To address these problems, various self-healing strategies have been developed,^6–8^ including approaches that leverage covalent^9–11^ and noncovalent^12,13^ dynamic bonds.

Supramolecular chemistry is a versatile tool for designing adaptive materials based on metal–ligand coordination,^14,15^ host–guest interactions,^16,17^ hydrogen bonding,^18,19^ and other noncovalent interactions that allow reversible (dis)­assembly^20–22^ in response to external stimuli. Materials designed using these principles exhibit functions that include self-healing,^12,23^ degradability,^24,25^ and shape memory behavior,^26,27^ also offering advantages in processing and recycling.^28,29^ Metallosupramolecular polymers (MSPs) have attracted considerable interest in this context.^30,31^ As the strength and dynamicity of the metal–ligand coordination can be widely tuned *via* the choice of the ligand and the metal salt, their properties can be readily adjusted.^32,33^ Many MSPs have been shown to exhibit excellent healability upon exposure to light or heat,^34–40^ including MSPs based on telechelic building blocks.^34–36^ These stimuli temporarily disassemble the MSP, reduce the molecular weight and viscosity, and thus facilitate flow and repair, before reassembly. However, many reported MSPs require *de novo* synthesis and often exhibit low mechanical strength, which limits their exploitability in real-world applications.^41,42^ Here we use a glycol-modified poly­(ethylene terephthalate) (PETG), which is attractive due to its mechanical robustness, transparency, chemical resistance, and processability,^43,44^ to demonstrate that healable MSPs can be conveniently accessed by postpolymerization modification of a commercial polymer. Previous work on supramolecular polyesters has focused on materials assembled with the hydrogen-bonding motif 2-ureido-4­[1*H*]-pyrimidinone (UPy) and demonstrated improved processability and reversible adhesion.^45–47^ However, their self-healing behavior remains unexplored. We show that the molecular weight of the telechelic building block is critical and identify a composition that combines efficient healing with high mechanical strength.

The stepwise synthesis of the macromonomers **M**_**xk**_ is shown in Scheme 1. To access PETG-based polyols, we first carried out a controlled depolymerization of commercial PETG *via* glycolysis with ethylene glycol in DMF at 100 °C, catalyzed by zinc acetate (Scheme S1). This transesterification reaction afforded hydroxyl-terminated PETG telechelics, whose molecular weight was controlled by the reaction time. Size-exclusion chromatography (SEC) reveals that the original PETG had a number-average molecular weight (*M*_n_) of ca. 30 kg mol^–1^, while the depolymerization products **PETG**_**xk**_ feature *M*_n_ values of *x* = 6 and 4 kg mol^–1^ (Figure S1, Table S1). ^1^H NMR spectra confirm the presence of the expected hydroxyl end groups (Figures S2–S5). Macromonomers were then synthesized by end-functionalizing the PETG telechelics with the well-known 2,6-bis­(1′-methyl-benzimidazolyl)­pyridine (Mebip) ligand,^26,32,34,36,42^ *via* a Mitsunobu reaction.^32,34^ SEC analysis confirms the successful covalent attachment of Mebip to the polymer chains, yielding macromonomers with *M*_n_ values of *x* = 9 and 6 kg mol^–1^, denoted as **M**_**xk**_. The dispersity (*Đ*) of these building blocks remained low, ranging from 1.3 to 1.5 (Table S1). A comparison of the elugrams acquired with the refractive index detector reveals that the molecular weight increase is largely due to the loss of lower-molecular-weight species upon functionalization, while the data acquired with a UV detector (346 nm), which selectively detects the Mebip-functionalized chains, provides evidence of end-group modification (Figure S1). The successful incorporation of the Mebip ligand at the polymer chain ends is corroborated by ^1^H NMR spectra (Supporting Figures S6–S9). The comparison of the ^19^F spectra of trifluoroacetic anhydride and hexafluoroacetone-derivatized **PETG**_**xk**_ and **M**_**xk**_ shows a high extent of functionalization (Supporting Figures S10–S11).^47,48^

**Scheme 1:**
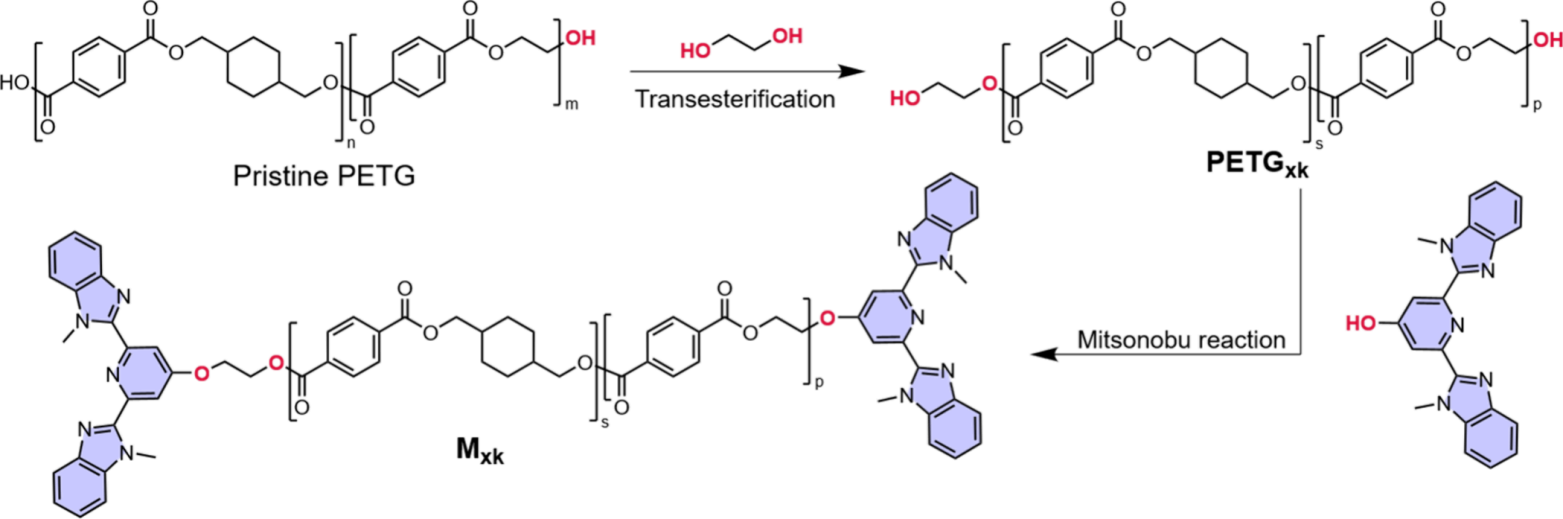
Synthesis of the Macromonomers **M**_**xk**_ *via* the Zn-Catalyzed Glycolysis of PETG, End-Functionalization of the Resulting Bifunctional Telechelics **PETG**_**xk**_ with the 2,6-Bis­(1′-methylbenzimidazolyl)­pyridine (Mebip) Ligand *via* the Mitsunobu Reaction

To investigate the complexation behavior of the macromonomers **M**_**xk**_ upon addition of Zn­(OTf)_2_ (Figure 1a) and to identify the ratio of these components at which full complexation is attained, spectrophotometric titrations conducted in CHCl_3_/CH_3_CN (9:1 v/v) were carried out. Figure 1b, which shows UV–vis absorption data for **M**_**6k**_, reveals that upon Zn^2+^ addition, the characteristic π → π* absorption band of the Mebip ligand at 314 nm progressively decreases, while a metal-to-ligand charge transfer (MLCT) band centered at 340 nm grows concomitantly. The analysis of the absorption intensity at 340 nm, plotted against the molar ratio of Zn­(OTf)_2_ to **M**_**6k**_, confirms that the complexation is complete at a Zn^2+^:**M**_**6k**_ molar ratio of 0.9:1 (Figure 1c). In the case of **M**_**9k**_, full coordination was achieved at a Zn^2+^:**M**_**9k**_ ratio of 1.3:1 (Figure S12). The deviation from a 1:1 ratio is likely related to uncertainties in the telechelics’ molecular weight (Table S1), but it may also indicate incomplete end-group functionalization.

**Figure 1:**
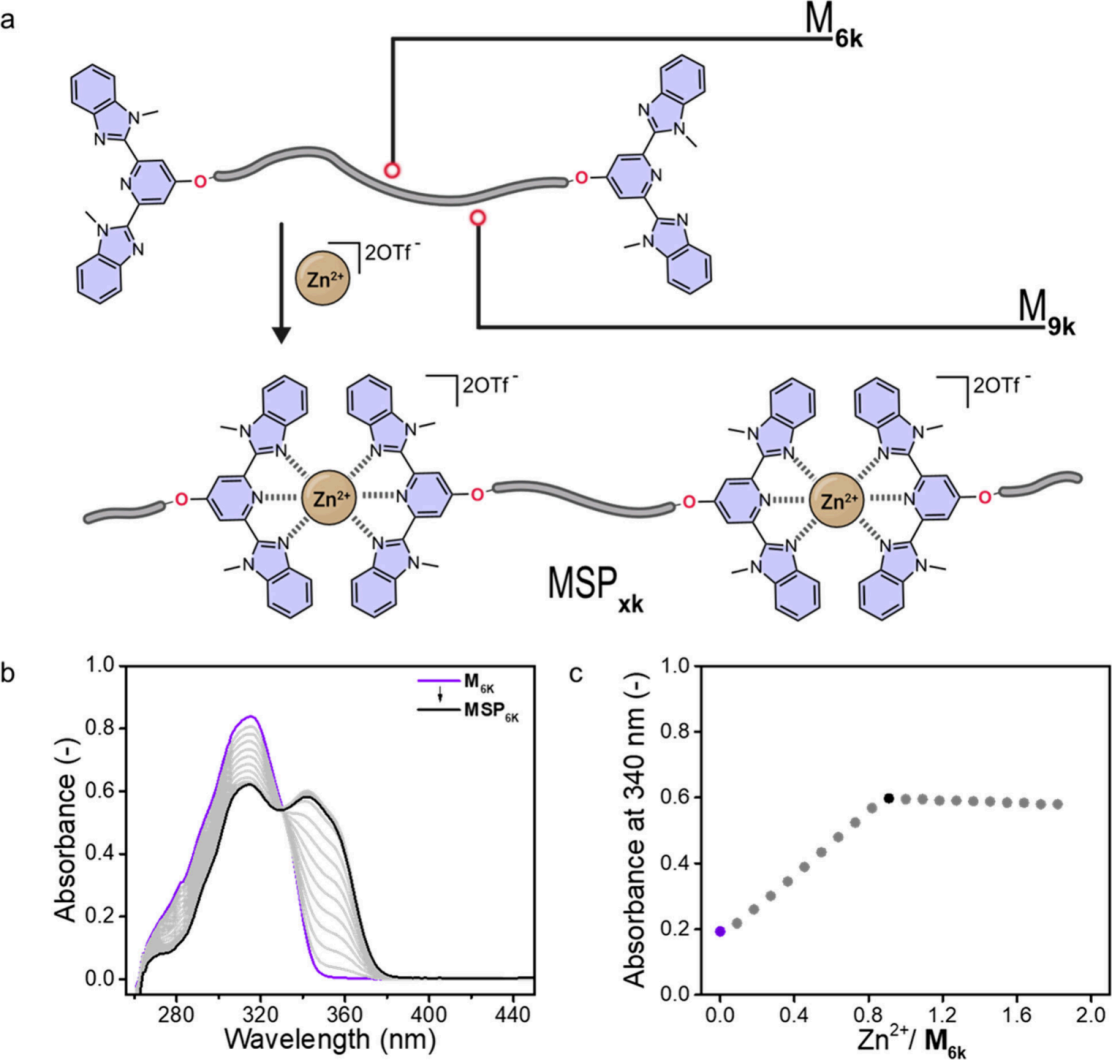
(a) Schematic representation of the complexation behavior of the macromonomers **M**_**xk**_ upon addition of Zn­(OTf)_2_. (b) UV–vis absorption spectra recorded during the titration of **M**_**6k**_ using *M*_n,NMR_ = 5316 g mol^–1^ (*c* = 230 μM) upon the addition of aliquots of Zn­(OTf)_2_ (*c* = 228 μM); the solvent was CHCl_3_/CH_3_CN 9:1 v/v The spectroscopic changes indicate the gradual transformation of the free ligand in **M**_**6k**_ (purple trace) into the metal–ligand complex that assembles **MSP**_**6k**_ (black trace). (c) Plot of the absorbance at 340 nm in the spectra shown in (b) (metal-to-ligand charge transfer band) against the Zn^2+^:**M**_**6k**_ ratio. The data shows that the complexation is complete at a Zn^2+^:**M**_**6k**_ ratio of 0.9:1.

**MSP**_**xk**_ films were prepared by dissolving **M**_**xk**_ in CHCl_3_/CH_3_CN and adding Zn­(OTf)_2_ until the absorption spectra of diluted aliquots indicated full complexation (see Supporting Information). The homogeneous solutions were then cast into poly­(tetrafluoroethylene) (PTFE) Petri dishes, and the solvent was evaporated before vacuum drying at 80 °C overnight, resulting in transparent, slightly purple films. As the processing history was found to matter (*vide infra*), the films were further reprocessed by compression-molding at 180 °C and rapid cooling. Gratifyingly, the UV–vis absorption spectra of **MSP**_**xk**_ films show, irrespective of the processing history, the characteristic MLCT band of the complex (Figure S13), which demonstrates that the supramolecular assembly occurs before the polymer reaches the glassy state.

The thermal properties of the original PETG, the hydroxy-terminated telechelics **PETG**_**xk**_, the macromonomers **M**_**xk**_, and **MSP**_**xk**_ were probed by thermogravimetric analysis (TGA) and differential scanning calorimetry (DSC). The TGA traces show a 5% mass loss around 380 °C for PETG and **PETG**_**xk**_; for **M**_**xk**_ and **MSP**_**xk**_ this temperature decreases only slightly to ca. 350 °C for the materials with the highest Mebip content (**M**_**6k**_ and **MSP**_**6k)**_ (Figures S14–S16, Table S2). Thus, like previously reported MSPs, the present materials display very high thermal stability.^47,49^

The first DSC heating traces of the solution-cast **MSP**_**xk**_ films show weak endothermic peaks between 100 and 180 °C, which are also observed in the DSCs of the solution-cast **PETG**_**xk**_ telechelics and the macromonomers **M**_**xk**_. We assign these events to the crystallization of PETG segments (Figure S17–S18, Table S2).^50,51^ These transitions are absent in the second heating traces of **MSP**_**xk**_, which instead only display a glass transition with a temperature (*T*_g_) of 92–93 °C. The fact that the *T*_g_ is slightly higher than that of PETG (83 °C) and the **PETG**_**xk**_ telechelics (66–72 °C) reflects the antiplasticizing nature of the metal–ligand (ML) complexes. The DSC traces (first and second heating) of the compression-molded samples only show a *T*_g_ and reflect that melt-processing affords amorphous solids. These findings are corroborated by wide-angle X-ray scattering (WAXS) data (Figure S19). While the diffractograms of the solution-cast films show well-defined, albeit weak, reflections, only amorphous halos are observable for the melt-processed samples. The small-angle X-ray scattering (SAXS) profile of solution-cast PETG (Figure S20a) shows a broad peak centered at q* = ca. 0.06 Å^–1^ that we ascribe to the crystalline phase of this material. Solution-cast **MSP**_**6k**_ also displays the same feature, albeit less pronounced, while **MSP**_**9k**_ displays only a small protuberance at q* = ca. 0.1 Å^–1^. This agrees with the WAXS data, which suggests a lower crystallinity for **MSP**_**9k**_ than for **MSP**_**6k**_. The lack of crystallinity after compression molding is also reflected in the SAXS profiles (Figure S20b). The SAXS plots of compression-molded **MSP**_**xk**_ films show an unpronounced peak at q* = ca. 0.1 Å^–1^, and a seemingly higher order reflection at *q* = ca. 0.15 Å^–1^, which may be indicative of weak and irregular phase separation of the ML complexes. Indeed, the Bragg peaks match those reported for Zn-based MSPs based on other telechelic building blocks (q* = 0.075–0.09 Å^–1^), which show well-defined lamellar morphologies.^52,53^ However, the extent of microphase separation in **MSP**_**9k**_ and **MSP**_**6k**_ is limited, which is in agreement with the lack of a melting transition of a ML hard phase in the DSC traces.

Dynamic mechanical analysis (DMA) measurements of compression-molded films provide more refined information on the thermal behavior and indirectly the morphology of the different MSPs (Figure 2a, Table S3). The DMA trace of PETG displays a glassy regime with a rather constant storage modulus *E*′ (1.0 GPa at 25 °C). *E*′ sharply drops around the *T*_g_ (86 °C, tan δ peak) and the sample fails at 111 °C. The DMA traces of **MSP**_**6k**_ and **MSP**_**9k**_ show slightly higher *T*_g_ values than established by DSC and reveal a rubbery plateau, which increases the failure temperature to ca. 155 °C. This behavior is diagnostic of microphase separation and the formation of a ML hard phase that physically cross-links the MSPs; however, the lower failure temperature and the lower modulus at the end of the rubbery plateau, compared to previous reports, suggest limited phase separation, in accordance with SAXS data.^34,42,52–55^

**Figure 2:**
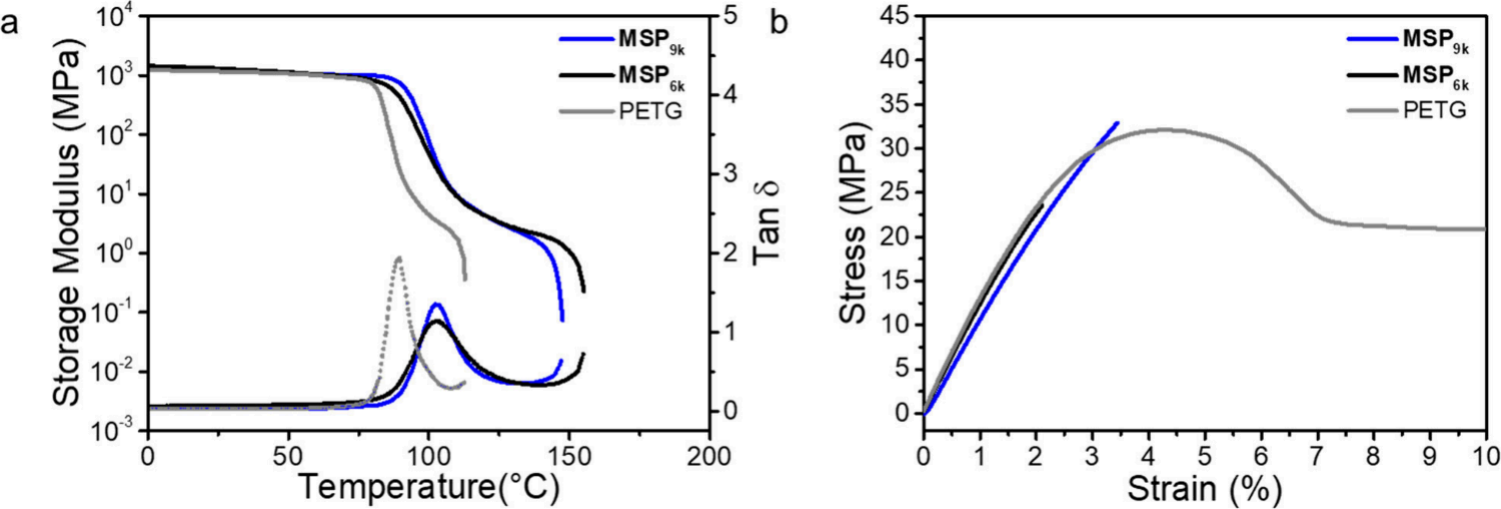
(a) Dynamic mechanical analysis (DMA) traces and (b) stress–strain curves of PETG and the **MSP**_**xk**_ films. The DMA data show the storage modulus (solid lines) and tan δ (dotted lines). The tensile tests were carried out at 25 °C and a strain rate of 150% min^–1^.

We further subjected the compression-molded samples to uniaxial tensile tests, which were carried out at 25 °C and a strain rate of 150% min^–1^ (Figure 2b, Table S3). The stress–strain curve of PETG shows a linear elastic regime with a Young’s modulus of ca. 1.4 GPa, a yield point at ca. 4% strain, and a yield stress of 36 MPa, followed by a plastic deformation regime and failure at a strain of ca. 260%. While **MSP**_**6k**_ is rather brittle and displays a substantially lower maximum stress (18 MPa) and elongation at break (2%) than PETG, **MSP**_**9k**_ displays a maximum stress (31 MPa) that is only slightly lower than that of PETG, and the brittleness is reduced in comparison to **MSP**_**6k**_ (failure strain = 3%). We speculate that the increased brittleness vis-à-vis PETG is primarily related to the inhomogeneities that the microphase separation imparts.^52,54,56^

In previous studies, a strong correlation was observed between the presence of a *G*′–*G*″ crossover point and the healing behavior of metallosupramolecular polymers (MSPs).^36,40,57^ The viscoelastic properties of the different MSPs were thus further probed by temperature-dependent oscillatory shear rheology measurements in the linear viscoelastic regime (Figure 3). The temperature sweep of PETG shows the typical behavior of a linear, entangled polymer above *T*_g_ (Figure 3a). At 100 °C, the material is in the rubbery regime with storage (*G*′) and loss (*G*′′) moduli of ca. 10^6^ and 3·10^5^ MPa, respectively. *G*′ and *G*′′ decrease with increasing temperature, a crossover point is seen at ca. 140 °C, and a terminal *G*′ value of ca. 10^4^ Pa is observed at 180 °C. As indicated by DSC and DMA data, **MSP**_**9k**_ and **MSP**_**6k**_ exhibit higher *T*_g_ values (90 and 95 °C, respectively, measured by DSC), which is reflected in the fact that at 100 °C, *G*′′ > *G*′. First crossover points that indicate the onset of the rubbery plateau are observed at ca. 107 °C (**MSP**_**9k**_) and ca. 112 °C (**MSP**_**6k**_) (Figure 3b,c). Especially the temperature sweep of **MSP**_**9k**_ shows a broad rubbery plateau, with a crossover point at 163 °C. The latter is associated with a slope change in the *G*’ trace, which we interpret as dissociation of the microphase-separated ML hard phase^54^ coupled with terminal relaxation of the polymer chains. The data of **MSP**_**6k**_ show a similar picture, although the crossover occurs at a slightly lower temperature (150 °C) and the rubbery plateau is less pronounced. We speculate that this may be related to the low molecular weight of the macromonomers and the increased molecular mobility of the dissociated species. Thus, the rheological behavior of the two MSPs is quite different from PETG and is (aside from the slightly higher *T*_g_) significantly influenced by the presence of physical cross-links.^58,59^

**Figure 3:**
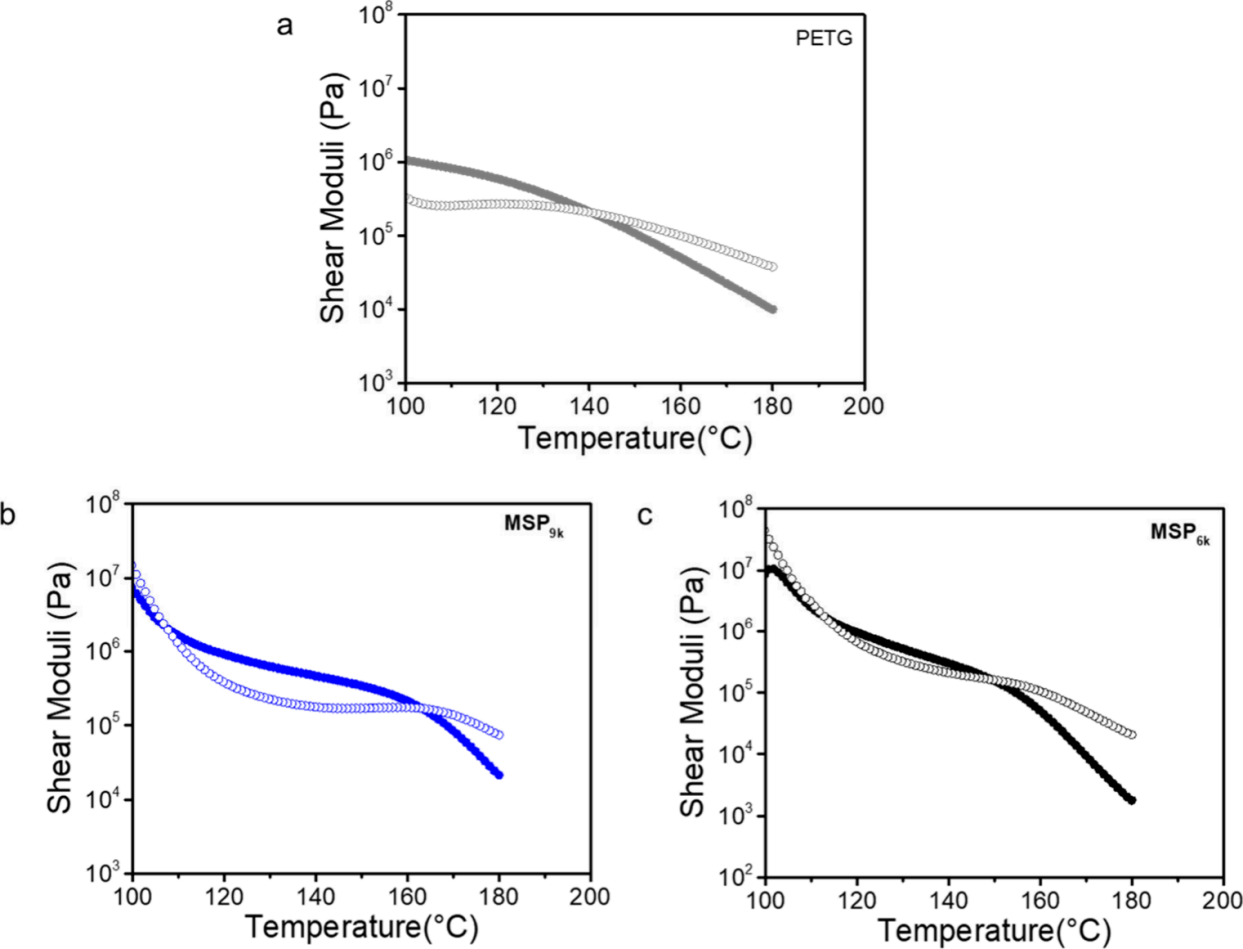
Temperature-dependent oscillatory shear rheology data of (a) PETG, (b) **MSP**_**9k**_, (c) **MSP**_**6k**_. Shown are storage moduli *G*′ (solid circles) and loss moduli *G*″ (open circles) as a function of temperature recorded at a fixed oscillatory frequency of ω = 10 rad·s^–1^, a constant strain of γ = 1% and a cooling rate of 3 °C min^–1^ from 180 to 100 °C.

A more in-depth rheological analysis was performed for **MSP**_**9k**_, with small-angle frequency sweeps performed at 180, 160, 140, and 120 °C with angular frequency (ω) varying from 0.1 to 100 rad·s^–1^ (Figure S21). At 160 °C, a crossover point at ca. 9 rad·s^–1^ is found, indicating a terminal relaxation time (τ_t_ = 2π/ω) of around 0.7 s. Time–temperature superposition of the frequency sweeps and fitting the Arrhenius law to the shift factors affords an activation energy (*E*_a_) of ca. 220 kJ mol^–1^. This value is slightly higher than that of other phase-segregated supramolecular polymers,^36,52,57,60–62^ and reflects a strong temperature-dependence of the viscoelastic properties of **MSP**_**9k**_. This is indicative of the interplay between PETG chain dynamics and phase separation, and reflects that there may be a small temperature window in which efficient healing is possible without melting the MSP.

We first probed the ability to heal the MSPs by qualitative experiments, in which films were cut to a depth of ca. 30% of their total thickness. The damaged samples were then subjected to heat treatment at 160 °C, which is near the crossover point of the two **MSP**_**xk**__,_ on a temperature-controlled stage, and the healing process was monitored by optical microscopy. In contrast to PETG, which could not be healed, **MSP**_**6k**_ and **MSP**_**9k**_ demonstrated rapid and complete healing; the cuts were no longer visible after 2 and 2.5 min, respectively (Figures S22–S24). Importantly, although the healing temperature exceeds the *T*_g_ values of **MSP**_**xk**_, the films retain their structural integrity, behaving as rubbery solids that rapidly heal due to the physical cross-links.

In view of the brittleness of **MSP**_**6k**_, quantitative healing experiments were only performed with films of **MSP**_**9k**_. Samples were cut and healed in the same manner as above. The healing efficacy was assessed by uniaxial tensile deformation experiments of original, damaged, and healed films. Figure 4a shows representative stress–strain curves, which clearly show the recovery of mechanical performance after healing. A comparison of the mechanical properties of the healed and the original samples reveals a healing efficiency of 81% with respect to the toughness and a recovery of 94% of the tensile strength (Figure 4b, Figure S22 and Table S4). The loss in toughness suggests the presence of residual defects that promote crack formation/propagation, decreasing the elongation at break.

**Figure 4:**
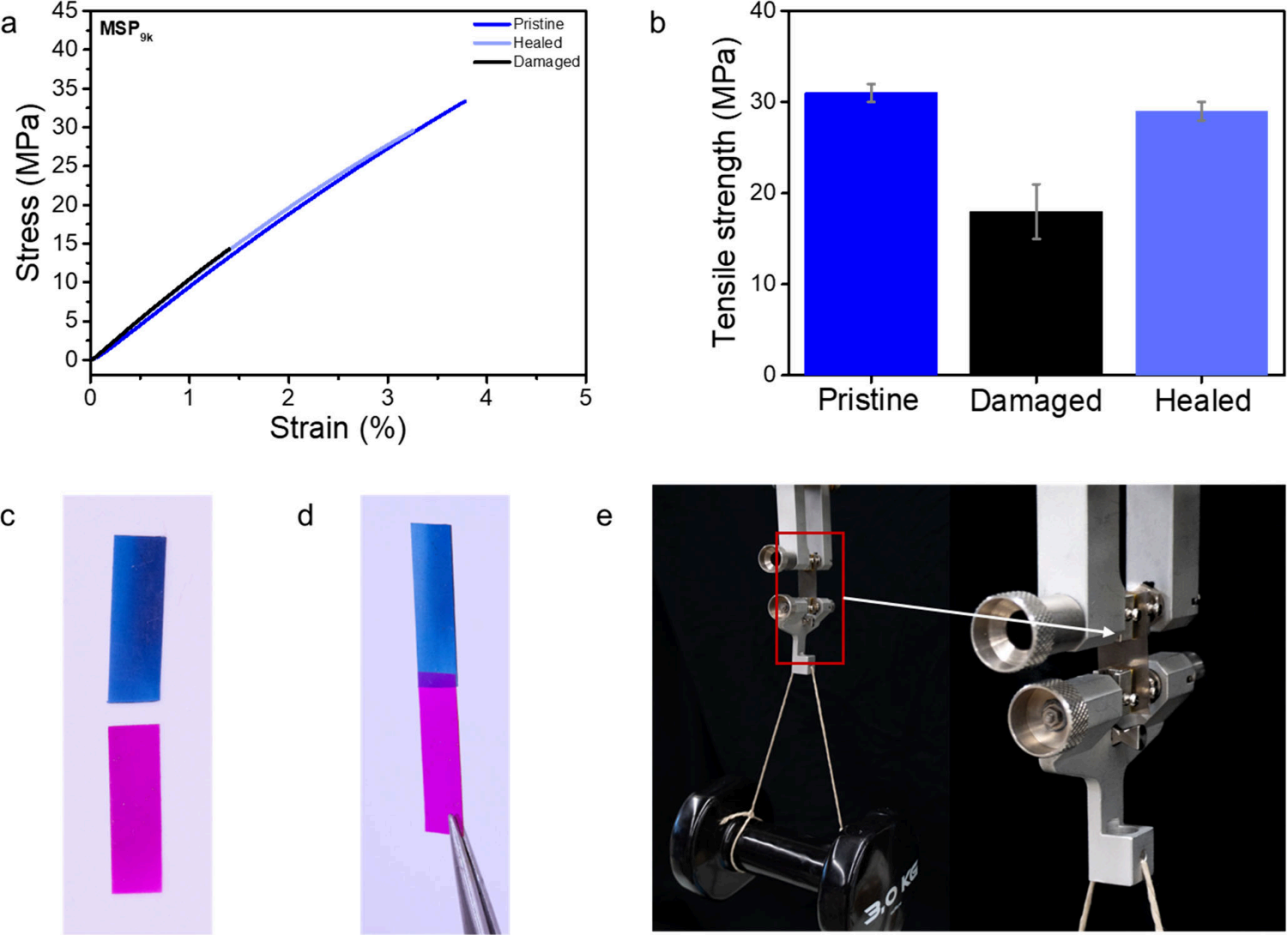
(a) Representative stress–strain curves of pristine, damaged, and healed **MSP**_**9k**_ samples were recorded at 25 °C. (b) Comparison of the tensile strength of pristine, damaged, and healed **MSP**_**9k**_ samples. Photograph of dyed pink and blue **MSP**_**9k**_ films before (c) and after (d) welding at 160 °C for 7 min. (e) Photograph of pristine **MSP**_**9k**_ film carrying a 3 kg dumbbell.

The dynamic properties of **MSP**_**9k**_ were further exploited to weld two films (Figure 4c–d). Uniaxial tensile tests reveal that the welded joint is slightly weakened compared to the pristine material, possibly due to the introduction of defects during the welding process (Figure S25 and Table S5). However, all samples showed cohesive failure, demonstrating the formation of a strong interface between the MSP films.^42^ Furthermore, the **MSP**_**9k**_ (200 μm thick) film exhibits remarkable mechanical robustness, successfully supporting a 3 kg load without tearing (Figure 4e).

In conclusion, we introduced healable MSPs that are easily accessible by the straightforward modification of a widely used commercial polyester. With **MSP**_**9k**_, we identified a material that combines high tensile strength, high Young’s modulus, and excellent healability. Indeed, aside from the significantly reduced elongation at break, the supramolecular polymer displays mechanical properties that mirror those of the parent PETG and appears to outperform known MSPs in its combination of healing efficiency, strength, and processability. However, combining these features with a higher extensibility remains a conundrum that may be hard to overcome. Indeed, it appears that the structural feature that limits this property, i.e., the presence of a ML hard phase, is also a considerable contributor to the material’s attractive properties. Further studies, including changing the metal cation and the counteranion,^63^ will aim at overcoming this limitation while still maintaining the high strength and rapid healing of the here reported materials.

## Supplementary Material



## Notes

Experimental procedures and characterization data, including ^1^H NMR spectra, SEC traces, additional titration data, TGA and DSC traces, X-ray diffractograms, rheology data, images that document the healing of damaged films, and tables with additional mechanical data (PDF)
