# Supplementary material for: Healable Glassy Metallosupramolecular Polymers
Source: ACS Macro Lett. 2025 Jun 30;14(7):996–1003. doi: 10.1021/acsmacrolett.5c00317 (PMC12269065; doi:10.1021/acsmacrolett.5c00317)
Supplement: Supplementary file 1 [file mz5c00317_si_001.pdf]

Supporting Information

Healable glassy metallosupramolecular polymers

Chaninya Mak-iad[1,2], Luca Bertossi[1], Georges J. M. Formon[1,2]*, Christoph Weder[1,2]*

[1]Adolphe Merkle Institute, University of Fribourg, Chemin des Verdiers 4, 1700 Fribourg, Switzerland

[2]NCCR Bio-inspired Materials, University of Fribourg, Chemin des Verdiers 4, 1700 Fribourg, Switzerland.

* georges.formon@unifr.ch; christoph.weder@unifr.ch

Experimental section

Materials

PETG (EASTAR 5011) was obtained from EASTMAN and dried at 80 °C in vacuo overnight before use. Zinc(II)trifluoromethanesulfonate ($Zn(OTf)_2$) was purchased from Strem Chemicals Inc. All other solvents and reagents were purchased from Sigma Aldrich or Acros and used without further purification.

Synthesis methods

2,6-Bis(1'-methylbenzimidazolyl)-pyridine (**Mebip-OH**) was prepared as reported in the literature.[1,2]

^{1}H NMR (300 MHz, DMSO-d6): δ 11.37 (s, 1H), 7.79 (s, 2H), 7.78 – 7.73 (m, 2H), 7.70 – 7.65 (m, 2H), 7.39 – 7.26 (m, 4H), 4.25 (s, 6H). 13C NMR (101 MHz, DMSO-d6): δ 165.1, 150.9, 149.9, 142.0, 137.0, 123.1, 122.3, 119.4, 112.4, 110.8, 32.5. HRMS (ESI): calcd. for $[M]^+$ 356.15 and $[M + Na]^+$ 378.13, found: 356.0 and 378.0.

Synthesis of bifunctional telechelics PETG$_{xk}$. In three separate three-necked round bottom flasks equipped with magnetic stir bars, PETG (2.00 g), ethylene glycol (0.10 g, 5 wt%), and $ZnAc_2$ (0.01 g, 0.5 wt%) were dissolved in DMF (30 mL) and the mixtures were stirred at 100 °C for either 1 h, or 3 h under N_2 to prepare **PETG$_{6k}$**, and **PETG$_{4k}$**, respectively. The solvent was evaporated, and the solid residues were separately dissolved in $CHCl_3$ (5 mL) and precipitated into MeOH (100 mL). The resulting solids were filtered off and dried for 24 hours in vacuo, yielding the respective **PETG$_{xk}$** as white solids (80%−85%).

PETG$_{6k}$: SEC (THF, PS standard): M_n = 6047 g mol^{-1}, $Đ$ = 1.7, ^{1}H NMR (400 MHz, Chloroform-d) δ 8.13 – 8.01 (m, 20H), 4.69 (d, 12H), 4.49 (dt, 1H), 4.29 (dd, 2H), 4.22 – 4.15 (m, 4H), 3.97 (s, 1H), 2.06 (s, 1H), 1.94 (d, 4H), 1.64 (q, 6H), 1.16 (d, 4H).

PETG$_{4k}$: SEC (THF, PS standard): M_n = 4275 g mol^{-1}, $Đ$ = 1.5 ^{1}H NMR (400 MHz, Chloroform-d) δ 8.09 (d, 22H), 4.69 (t, 13H), 4.53 – 4.44 (m, 2H), 4.29 (dt, 2H), 4.19 (dt, 5H), 3.98 (dd, 2H), 2.06 (s, 1H), 1.95 – 1.90 (m, 4H), 1.81 (s, 2H), 1.57 (s, 2H), 1.16 (d, 4H).

Synthesis of telechelic macromonomers M$_{xk}$. PETG$_{4k}$ (1.0 g, 0.23 mmol of OH groups) was dried under a high vacuum overnight. A two-necked round-bottom flask equipped with a magnetic stir bar was charged with 20 mL of anhydrous THF under N_2, and the mixture was stirred until a

homogeneous solution had formed. **Mebip-OH** (0.5 g, 1.4 mmol), triphenylphosphine (0.906 g, 3.5 mmol), and anhydrous THF (20 mL) were added, and the mixture was cooled in an ice/NaCl bath to −10 °C and stirred. When all reactants had dissolved, a 40 wt% diethyl azo-dicarboxylate (DEAD) solution in toluene (2 mL) was added dropwise under constant stirring. The cooling bath was removed after 4 h, and the reaction mixture was stirred at ambient temperature for another 24 h, before it was precipitated in acetone (400 mL). The purple product, $\mathbf{M_{6k}}$, was collected by filtration and dried under high vacuum at 65 °C for 2 days. Macromonomer $\mathbf{M_{9k}}$ was synthesized following the same procedure using $\mathbf{PETG_{6k}}$ (1.00 g, 0.17 mmol), **Mebip-OH** (0.22 g, 0.63 mmol), triphenylphosphine (0.42 g, 1.62 mmol), and DEAD solution (1 mL). The crude products were purified by column chromatography ($CHCl_3$: MeOH 95:5), yielding pink solids (65−68%).

$\mathbf{M_{9k}}$: SEC (THF, PS standard): M_n = 8668 g mol^{-1}, Ð = 1.3, ^{1}H NMR (400 MHz, Chloroform-d): Mn = 7492 g mol^{-1}, δ 8.12 (dd, 31H), 8.03 (d, 1H), 7.87 (dt, 1H), 7.52 – 7.31 (m, 3H), 4.84 – 4.76 (m, 1H), 4.72 (t, 21H), 4.57 – 4.48 (m, 1H), 4.31 (d, 3H), 4.26 (s, 3H), 4.21 (s, 7H), 4.06 – 3.89 (m, 1H), 2.08 (d, 2H), 1.96 (d, 7H), 1.83 (s, 4H), 1.61 (d, 9H), 1.28 (s, 1H), 1.18 (s, 7H).
$\mathbf{M_{6k}}$: SEC (THF, PS standard): M_n = 6118 g mol^{-1}, $Đ$ = 1.3, ^{1}H NMR (400 MHz, Chloroform-d): Mn = 5316 g mol-1, δ 8.09 (s, 22H), 7.91 (d, 1H), 7.52 – 7.34 (m, 3H), 4.78 (d, 1H), 4.68 (d, 14H), 4.50 (d, 1H), 4.32 – 4.23 (m, 5H), 4.22 – 4.15 (m, 5H), 3.93 – 3.72 (m, 1H), 2.16 (s, 2H), 2.06 (s, 1H), 1.94 (d, 5H), 1.85 (q, 0H), 1.72 – 1.46 (m, 3H), 1.17 (d, 5H), 0.07 (s, 1H).

Spectrophotometric titration of $\mathbf{M_{xk}}$. UV-vis absorption spectra were recorded during the incremental addition of a $Zn(OTf)_2$ solution (c = 228 μM) in CH_3CN to a solution of $\mathbf{M_{6k}}$ (c = 12.6 μM, M_n = 5316 g mol^{-1} calculated from ^{1}H NMR spectroscopy) in $CHCl_3/CH_3CN$ 9:1 for the formation of the $\mathbf{MSP_{6k}}$ complex. The decrease in the characteristic absorption band of the Mebip ligand at 314 nm was accompanied by an increase in the MLCT band at approximately 340 nm upon the addition of $Zn(OTf)_2$ aliquots indicating coordination. The titration of $\mathbf{M_{9k}}$ (c = 12.6 μM, M_n = 7492 g mol^{-1} calculated from ^{1}H NMR spectroscopy) was performed following an analogous procedure.

Synthesis of $\mathbf{MSP_{xk}}$. $\mathbf{M_{6k}}$ (0.10 g) was dissolved in $CHCl_3/CH_3CN$ 9:1 (1 mL), and a solution of $Zn(OTf)_2$ (0.057 M) in CH_3CN was added in portions. To ensure complete complex formation, the complexation was monitored by UV-vis spectroscopy by taking 2 μL aliquots of the mixture after each addition of $Zn(OTf)_2$. Initially, half of the stoichiometric amount of $Zn(OTf)_2$ was added,

followed by the gradual addition of the remaining $Zn(OTf)_2$ solution to the polymer stock solution until full complexation was confirmed by UV-vis absorption spectroscopy. The MSP solution was then cast into a poly(tetrafluoroethylene) (PTFE) Petri dish with a diameter of 4 cm, and the solvent was evaporated under ambient conditions in a well-ventilated hood. The resulting $\mathbf{MSP_{6k}}$ film was further dried under a vacuum at 80 °C overnight. The same procedure was followed for the preparation of $\mathbf{MSP_{9k}}$.

Preparation of compression-molded films. The solution-cast $\mathbf{MSP_{xk}}$ films were optionally re-processed by compression-molding in a model 3851-0 Carver press. The films were placed between two PTFE sheets separated by 100 µm thick spacers for scratch healing tests and 200 µm thick spacers for all other characterizations and compression-molded at 180 °C under a pressure of 6 tons for 2 min. The films (still between the PTFE sheets) were then carefully removed from the press and rapidly placed between two cold metal plates to ensure rapid cooling to room temperature. The $\mathbf{MSP_{xk}}$ films thus produced had a thickness of either ca. 100 or 200 µm.

Preparation of dyed, metallosupramolecular films for welding. A solution of $\mathbf{MSP_{9k}}$ was prepared at a concentration of 200 mg in 2 mL $CHCl_3/CH_3CN$ 9:1 . To this solution, rhodamine B (pink) was added to reach a final concentration of 0.1 wt% compared to the polymer. The same procedure was followed using indigo as the blue dye. Each dyed MSP solution was then cast into separate poly(tetrafluoroethylene) (PTFE) Petri dishes with a diameter of 4 cm, and the solvent was evaporated under ambient conditions in a well-ventilated hood. The resulting dyed $\mathbf{MSP_{9k}}$ films were further processed using the same protocol as undyed films (described above), reaching homogeneous pink and blue films of ca. 200 µm thickness. These films were cut into strips with a width of ca. 5mm and cut in half crosswise.

Characterization

Nuclear magnetic Resonance (NMR) spectroscopy. ^1H (400 MHz), ^{13}C (100 MHz), and ^{19}F NMR (282 MHz) spectra were recorded by a Bruker A VIII HD spectrometer in DMSO-d_6 or chloroform-d, and processed and analyzed using MestReNova software (version 11.0). Chemical shifts (δ) are reported in parts per million (ppm), with spectra calibrated against the residual solvent peak: $CDCl_3$ δ = 7.26 ppm for ^1H NMR and δ = 77.16 ppm for ^{13}C NMR; DMSO-d_6 δ = 2.50 ppm for ^1H NMR and δ = 39.52 ppm for ^{13}C NMR.[3] Coupling constants (J) are expressed in Hz

(multiplicity: s = singlet, bs= broad singlet, d= doublet, dd= doublet of doublets, t = triplet, q= quartet, m = multiplet, br = broad signal).

Identification of the functionalization of derivatized $\mathbf{PETG_{xk}}$ and $\mathbf{M_{xk}}$ by ^{19}F NMR spectroscopy. The samples of the hexafluoroacetone (HFA) adducts of the hydroxy groups in $\mathbf{PETG_{xk}}$ and $\mathbf{M_{xk}}$ were prepared by using a 10% HFA solution in chloroform-d, following a previously reported protocol.[4] Thus, 0.45 mL of the 10% HFA solution was added to a solution containing 0.025 mmol of the respective $\mathbf{PETG_{xk}}$ or $\mathbf{M_{xk}}$ in 0.5 mL of $CDCl_3$. Cyclohexanol (0.025 mmol) was included as an internal standard. In a separate procedure, trifluoroacetic anhydride (TFAA) adducts were prepared by adding one drop of TFAA to ca. 20 mg of the respective $\mathbf{PETG_{xk}}$ or $\mathbf{M_{xk}}$ in 0.5 mL of chloroform-d.

Size exclusion chromatography (SEC). SEC measurements were conducted on an Agilent Technologies 1200 series HPLC system equipped with an Agilent PLgel mixed guard column (particle size = 5 μm) and two Agilent PLgel mixed-D columns (7.5 mm ID × 300 mm L, particle size = 5 μm). THF was utilized as the eluent at a 1.0 mL min^{-1} flow rate. Detection was achieved using a UV detector (Agilent 1200 series, λ = 346 nm) and an interferometric refractometer detector (Agilent 1260). Data acquisition and processing were carried out using Agilent ChemStation, with molecular weights (M_n) and dispersity ($Đ$) determined relative to polystyrene (PS) standards.

UV-vis absorption spectroscopy. Spectra were acquired using a Shimadzu UV-2401 PC spectrophotometer in $CHCl_3/CH_3CN$ solvent mixtures. Measurements were conducted in quartz cuvettes with a 1 cm path length.

For UV-vis measurements on films, a 5 mM solution of $\mathbf{MSP_{9k}}$ was produced as described above, but instead of solvent casting, the solution was spin-coated on a quartz slide. After air drying inside a fume hood, the "solution-cast" coated slide was measured. The slide was then heated to 180 °C for 2 minutes with a hot plate, to mirror the compression-molding conditions, and was then: cooled rapidly by removing it from the hot plate, yielding the "rapid cooling" sample; or left on the hot plate to cool slowly, reaching the "normal cooling" sample. Spin-coated samples of PETG and $\mathbf{M_{9k}}$ were prepared analogously. The UV-vis of these thin films was measured using the PETG sample as a reference.

X-Ray scattering. Small-angle X-ray scattering (SAXS) and wide-angle X-ray scattering (WAXS) measurements were conducted using a NanoMax-IQ camera (Rigaku Innovative Technologies). The instrument was equipped with a Cu target-sealed tube source (λ = 1.54 Å) (MicroMax 003 microfocus, Rigaku), and scattering data were collected using a Pilatus 100 K detector (Dectris). Scattering data are presented as a function of the momentum transfer $q = (4\pi/\lambda)*\sin(2\theta/2)$, where θ is the scattering angle.

Thermogravimetric analysis (TGA). TGA measurements were performed under a nitrogen atmosphere using a Mettler-Toledo TGA/DSC 1 STAR system. Samples were heated from 25 to 600 °C at a rate of 10 °C min^{-1}.

Differential scanning calorimetry (DSC). DSC analyses were conducted under a nitrogen atmosphere using Mettler-Toledo DSC 2 and DSC 5+ STAR systems calibrated with high purity Indium and Zinc. The samples were subjected to heating and cooling cycles from −80 to 200 °C at a rate of 10 °C min^{-1}.

Dynamic mechanical analysis (DMA). DMA measurements were performed using a TA Instruments DMA Q800. Analyses were conducted with a heating rate of 3 °C min^{-1}, a frequency of 1 Hz, and an amplitude of 15 μm over a temperature range of −80 to 200 °C. Rectangular samples (width: 5.35 mm, thickness: 0.2 mm) were used. Reported mechanical properties represent the mean of 3–5 independent measurements, with standard deviations provided as error estimates.

Tensile testing. Tensile tests were performed at room temperature (25°C) in accordance with ASTM D882 using a Zwick/Roell static material testing machine equipped with a 200 N Xforce HP load cell. Rectangular samples (width: 5.35 mm, thickness: 200 μm) were tested at a strain rate of 150 % min^{-1}. Reported data represent the mean of 3–5 independent measurements, and errors quoted are standard deviations.

Rheology. Shear rheometry was conducted using an Anton Paar MCR 102e rheometer equipped with Peltier plates. A plate-plate geometry with a 25 mm diameter was utilized with a sample thickness of ca. 200 μm. Samples were positioned within the geometry and equilibrated for 10 minutes at 180 °C to ensure optimal contact with the plates.

Temperature sweep tests were performed over a temperature range of 180 to 100 °C at a cooling rate of 3 °C min^{-1}. The tests were conducted at a fixed oscillatory frequency of ω = 10 rad·s^{-1} and a constant strain of γ = 1%. The storage modulus (G') and loss modulus (G'') were measured and plotted as functions of temperature.

Frequency sweep experiments were conducted by initially heating the sample to 180 °C to ensure optimal contact with the rheometer plates. After a 10 min resting period to achieve thermal homogeneity, the experiment commenced. Subsequent temperatures (160, 140, and 120 °C) were attained with a cooling rate of 10 °C min^{-1}, followed by a 10 min equilibration period to maintain thermal homogeneity. Data were collected across angular frequencies ranging from 100 to 0.1 rad·s^{-1} at a constant strain of γ = 1%.

The master curve was constructed by applying the time-temperature superposition (TTS) principle. Frequency sweep experiments were shifted by manually adjusting the horizontal shift factors (a_T). Vertical shifting was not applied, as it is expected to remain relatively constant over the specified temperature range. A plot of ln(a_T) versus $1/T$ was generated; the slope of the linear fit to this plot was then multiplied by the universal gas constant (R) to calculate the activation energy (E_a).[5]

Scratch healing tests. To investigate the scratch healing behavior, thin films of PETG and $\mathbf{MSP_{xk}}$ with dimensions of ca. 10×5.35×0.10 mm (length × width × thickness) were prepared and scratched to a depth of ca. 30% of the original sample thickness using a razor blade attached to a caliper for precise depth control. A glass slide was placed on a temperature-controlled microscopy stage (Linkam, Linksys32 software) and pre-heated to 160°C. After reaching the desired temperature, the $\mathbf{MSP_{xk}}$ films were placed on top of the glass slide for ca. 2.5 min until the scratch disappeared. The process was monitored using the attached optical microscope (Olympus BX51 with a DP71 digital camera). Quantitative healing experiments were only performed with films of $\mathbf{MSP_{9k}}$. Samples were cut and healed by heat treatment in the same manner as above. The healing efficacy was assessed by uniaxial tensile deformation experiments of original, damaged, and healed films.

Welding of metallosupramolecular films. A pink and a blue $\mathbf{MSP_{9k}}$ film were overlapped with a 1-2 mm contact region and placed on top of a hot plate at 160°C for 1 min without any pressure, then 6 minutes with a weight of 100 g placed on top. The welded films were taken off the hot plate, cooled rapidly, and tested with uniaxial tensile testing.

Supplementary Figures, Scheme, and Tables

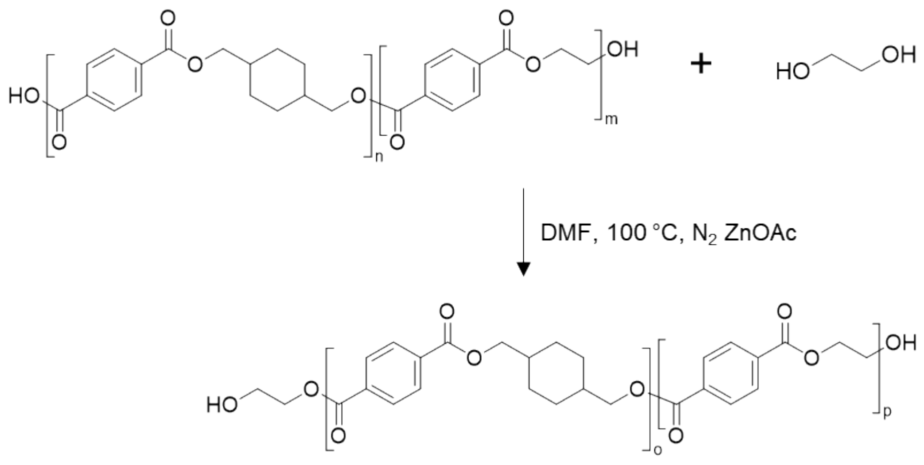

Scheme S1. Synthesis of the hydroxy-terminated telechelic PETGs (**PETG$_{xk}$**) series.

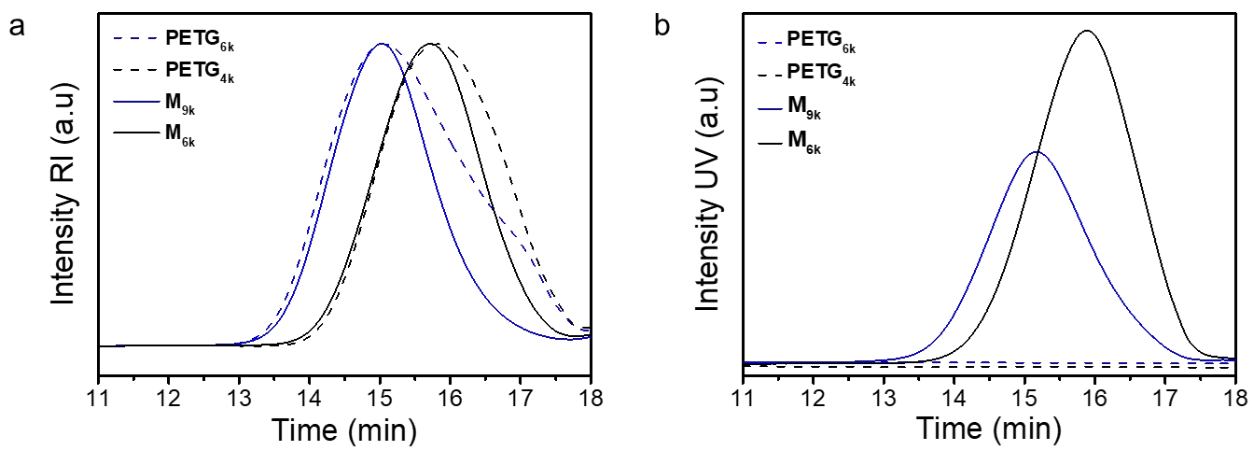

Figure S1. Representative size exclusion chromatography (SEC) elugrams of the hydroxy-terminated telechelics **PETG$_{xk}$** and of the corresponding macromonomers **M$_{xk}$**. (a) Elugrams recorded with the refractive index detector (RI), normalized to the maximum intensity of the main peak. (b) Elugrams recorded with the UV-detector (λ = 346 nm), demonstrating that in the macromonomers **M$_{xk}$** the Mebip ligand is covalently attached.

NMR Spectra

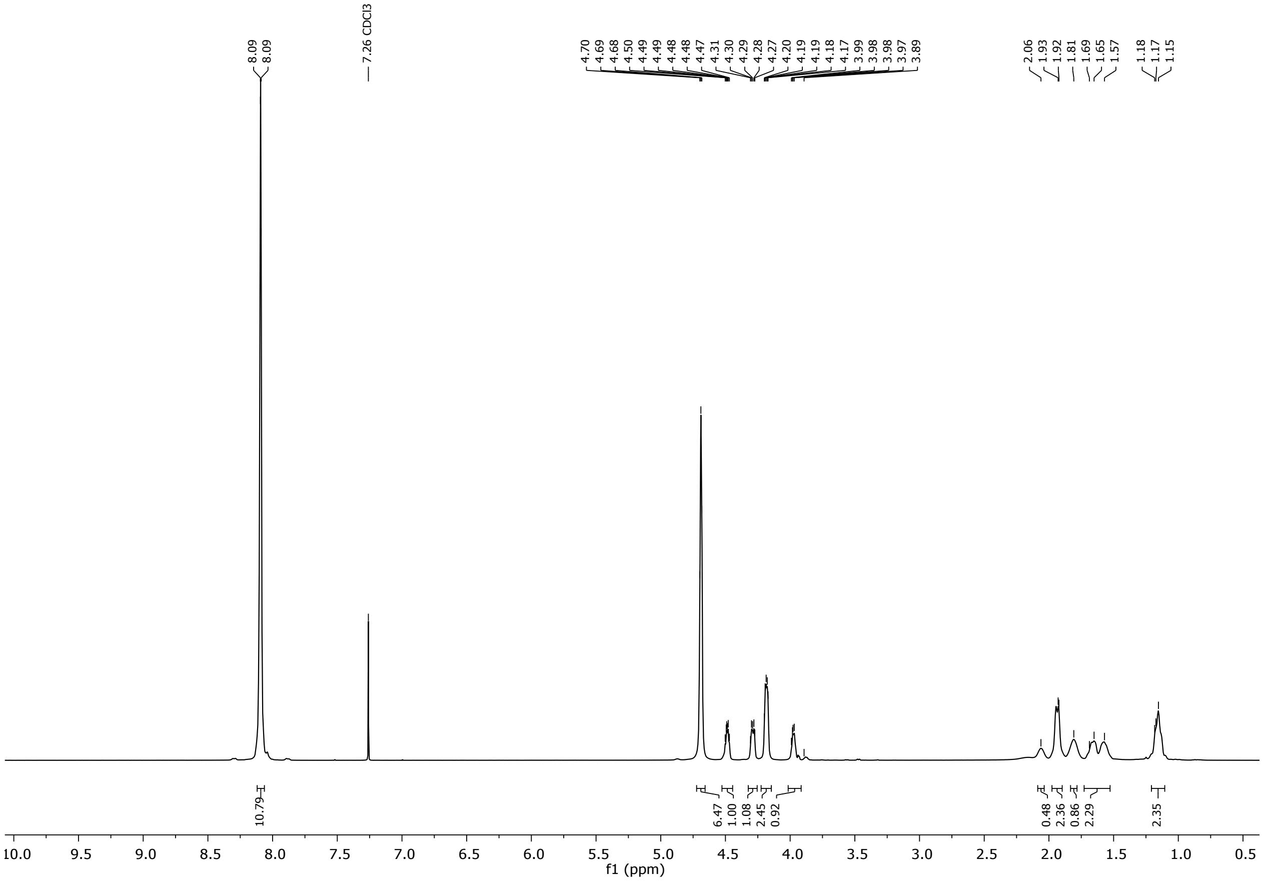

Figure S2. ^{1}H NMR Spectrum ($CDCl_3$, 400 MHz) of $\mathbf{PETG_{4k}}$.

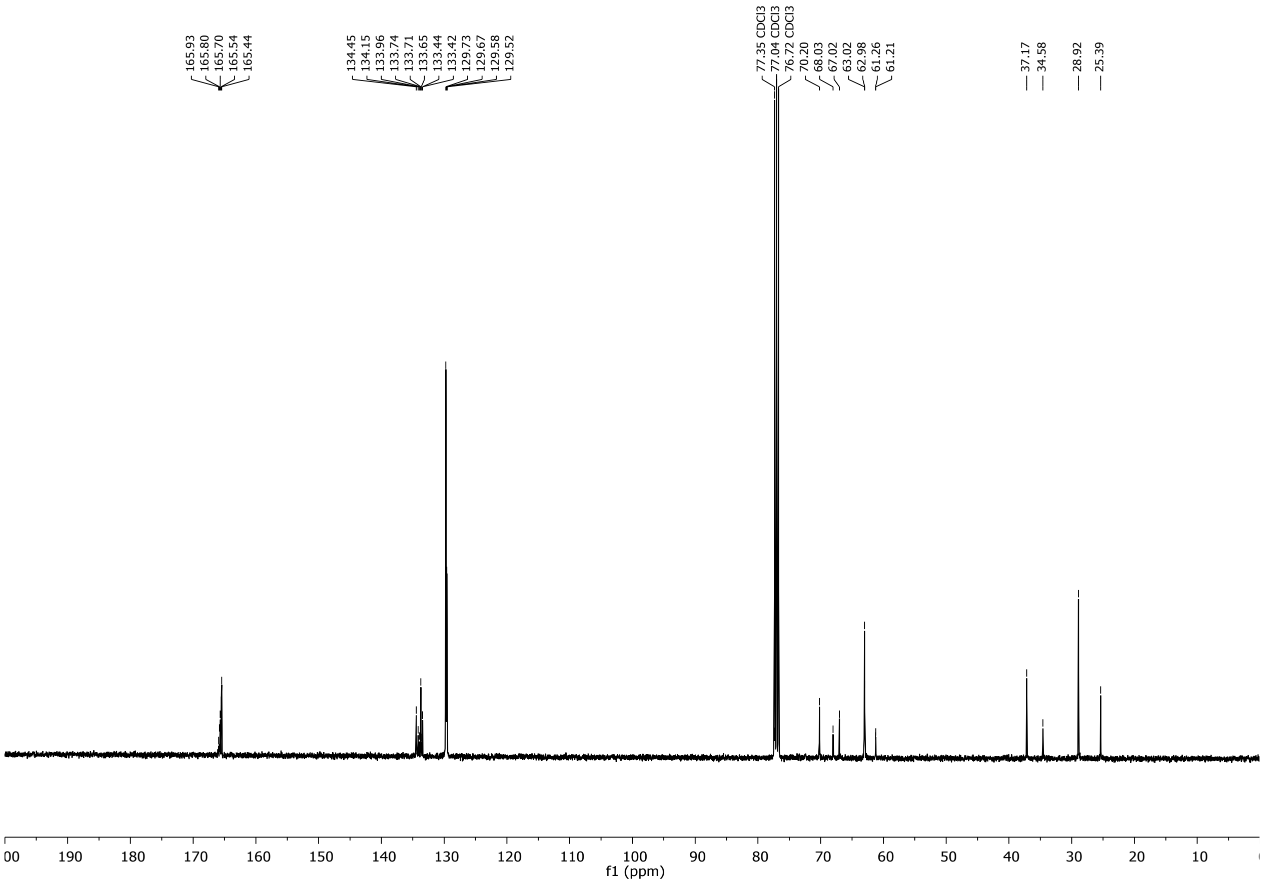

Figure S3. ^{13}C NMR spectrum ($CDCl_3$, 101 MHz) of **$PETG_{4k}$**.

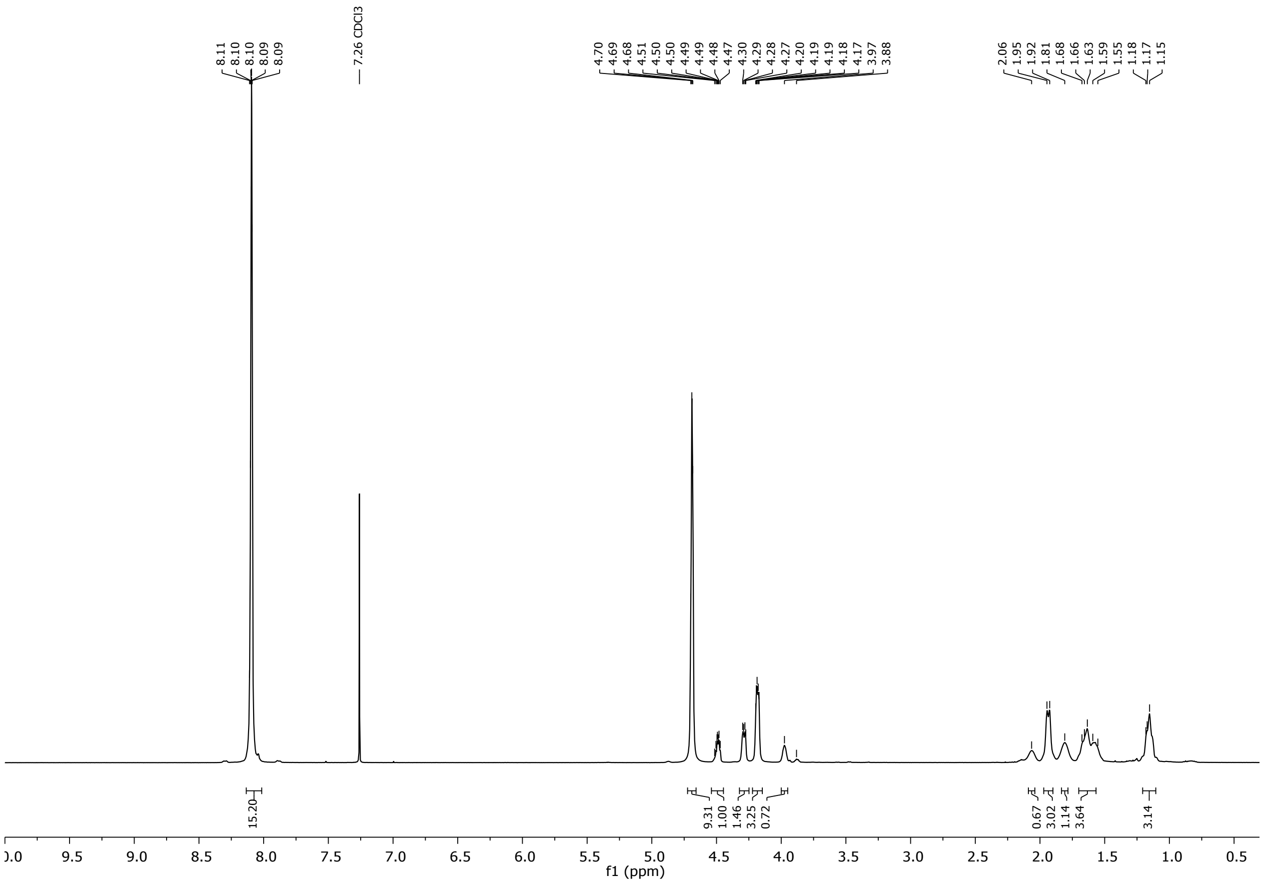

Figure S4. ^{1}H NMR Spectrum ($CDCl_3$, 400 MHz) of **PETG$_{6k}$**.

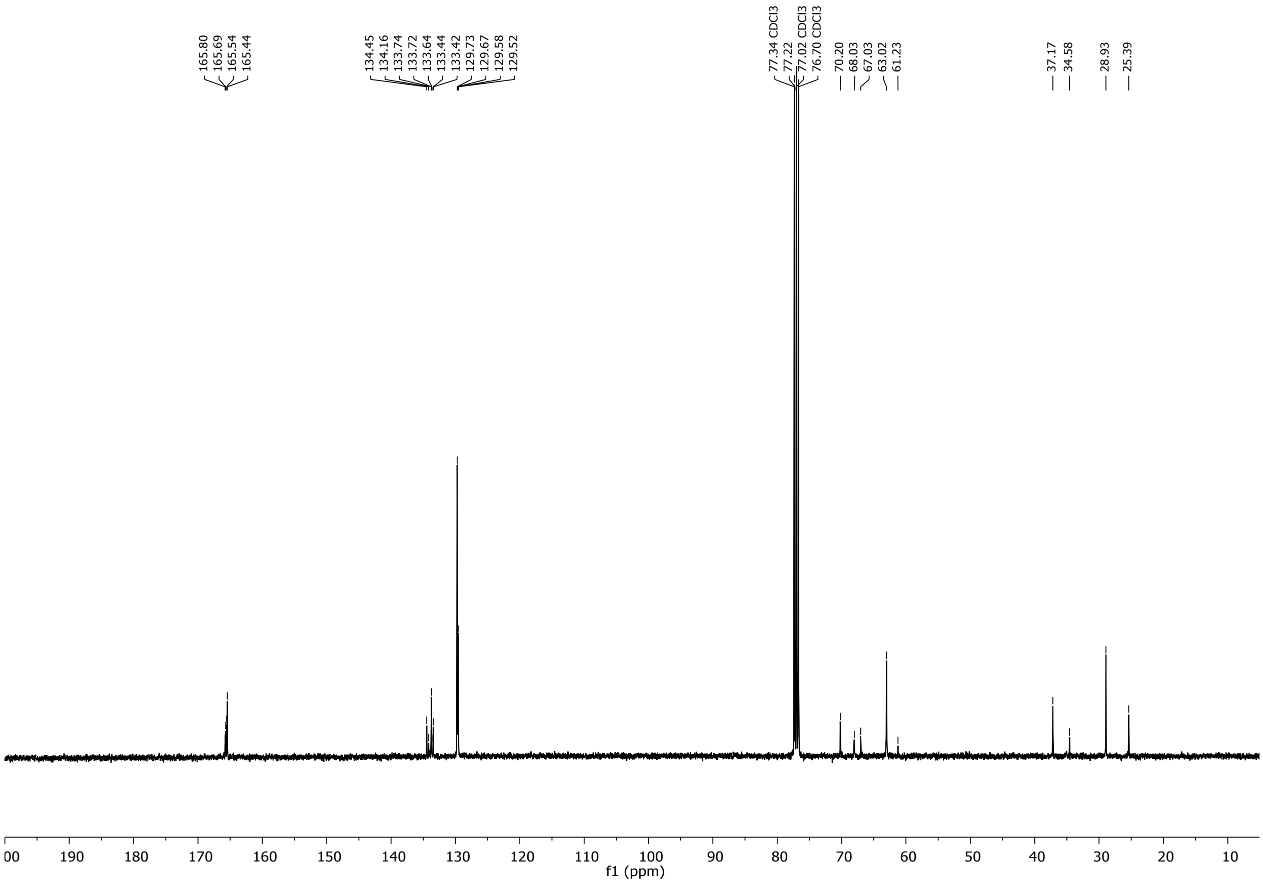

Figure S5. ^{13}C NMR spectrum ($CDCl_3$, 101 MHz) of $\mathbf{PETG_{6k}}$.

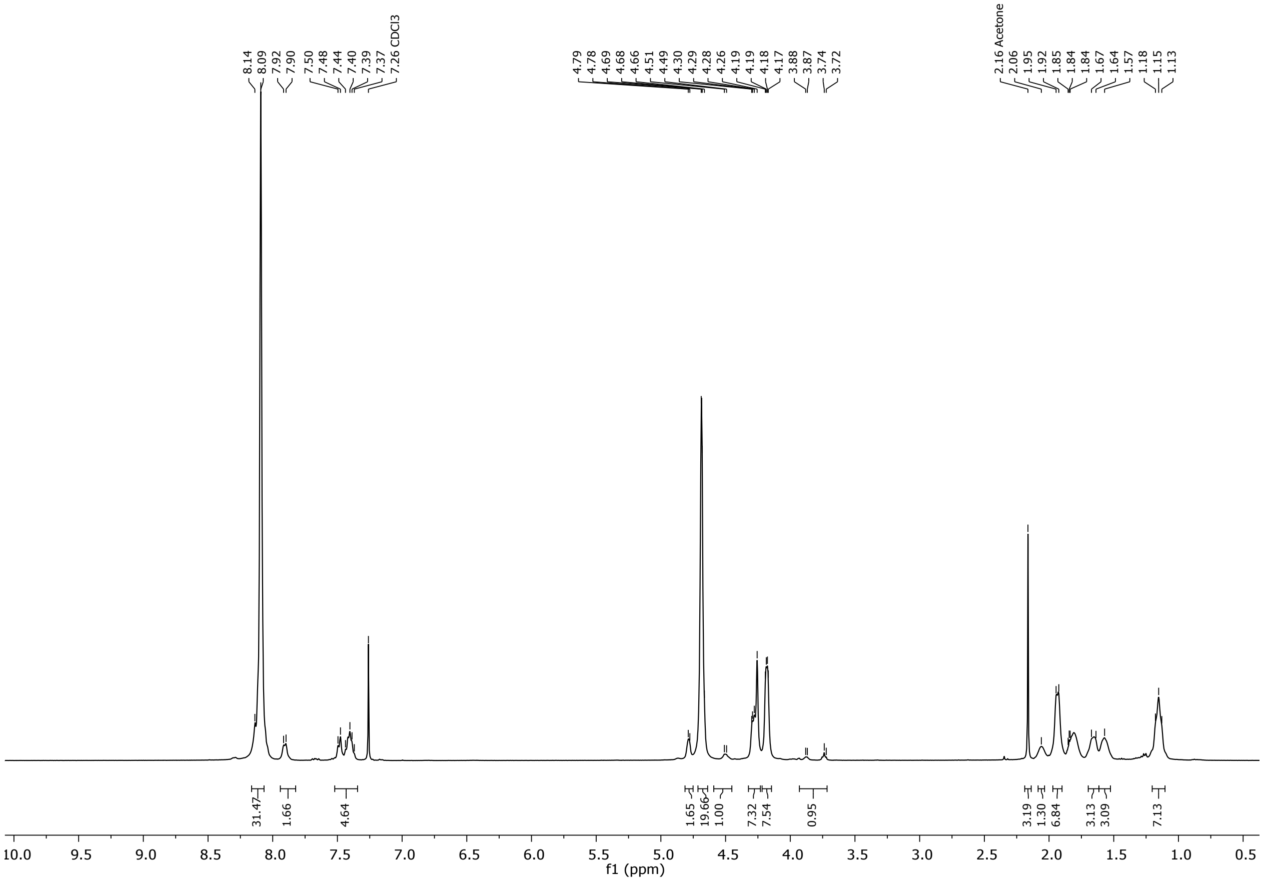

Figure S6. ^{1}H NMR Spectrum ($CDCl_3$, 400 MHz) of $\mathbf{M_{6k}}$.

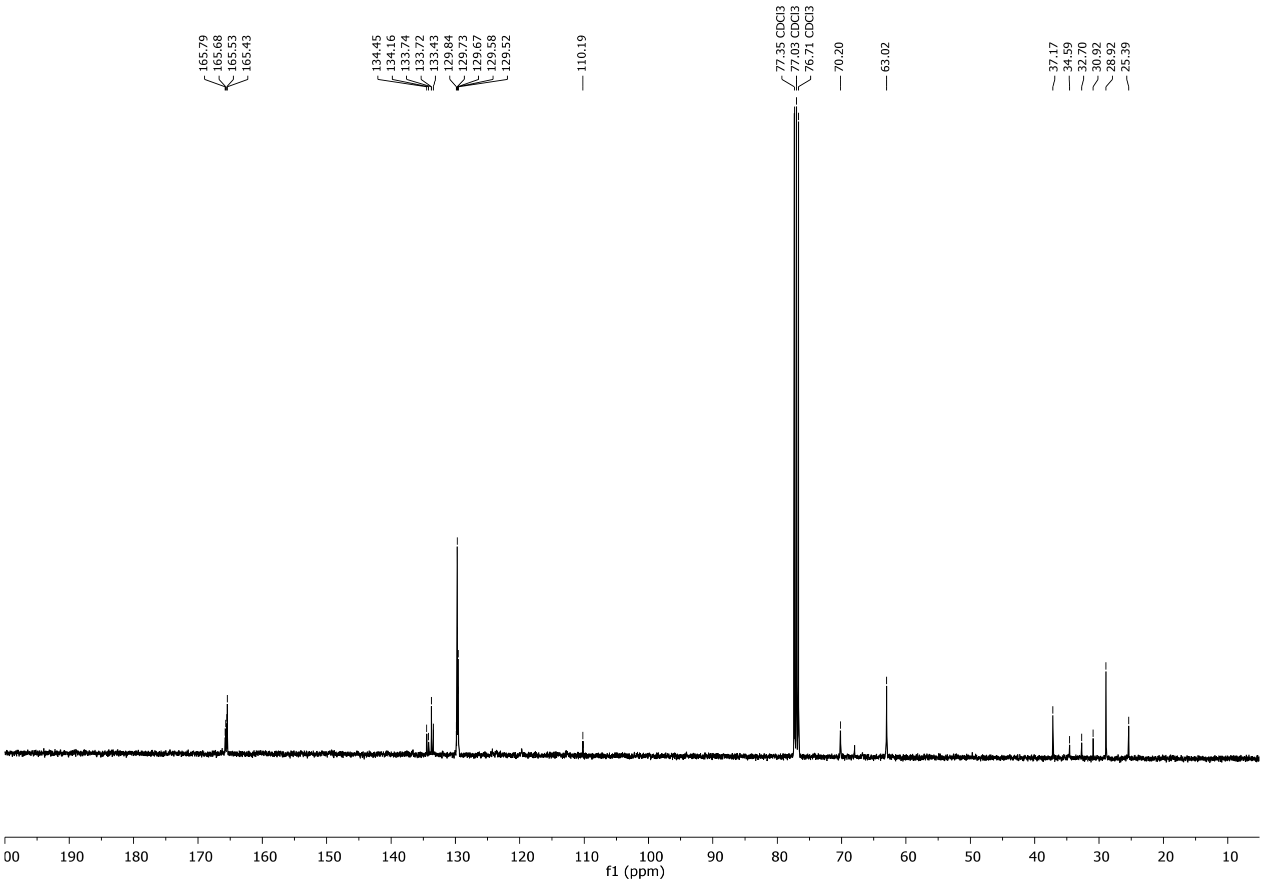

Figure S7. ^{13}C NMR spectrum ($CDCl_3$, 101 MHz) of $\mathbf{M_{6k}}$.

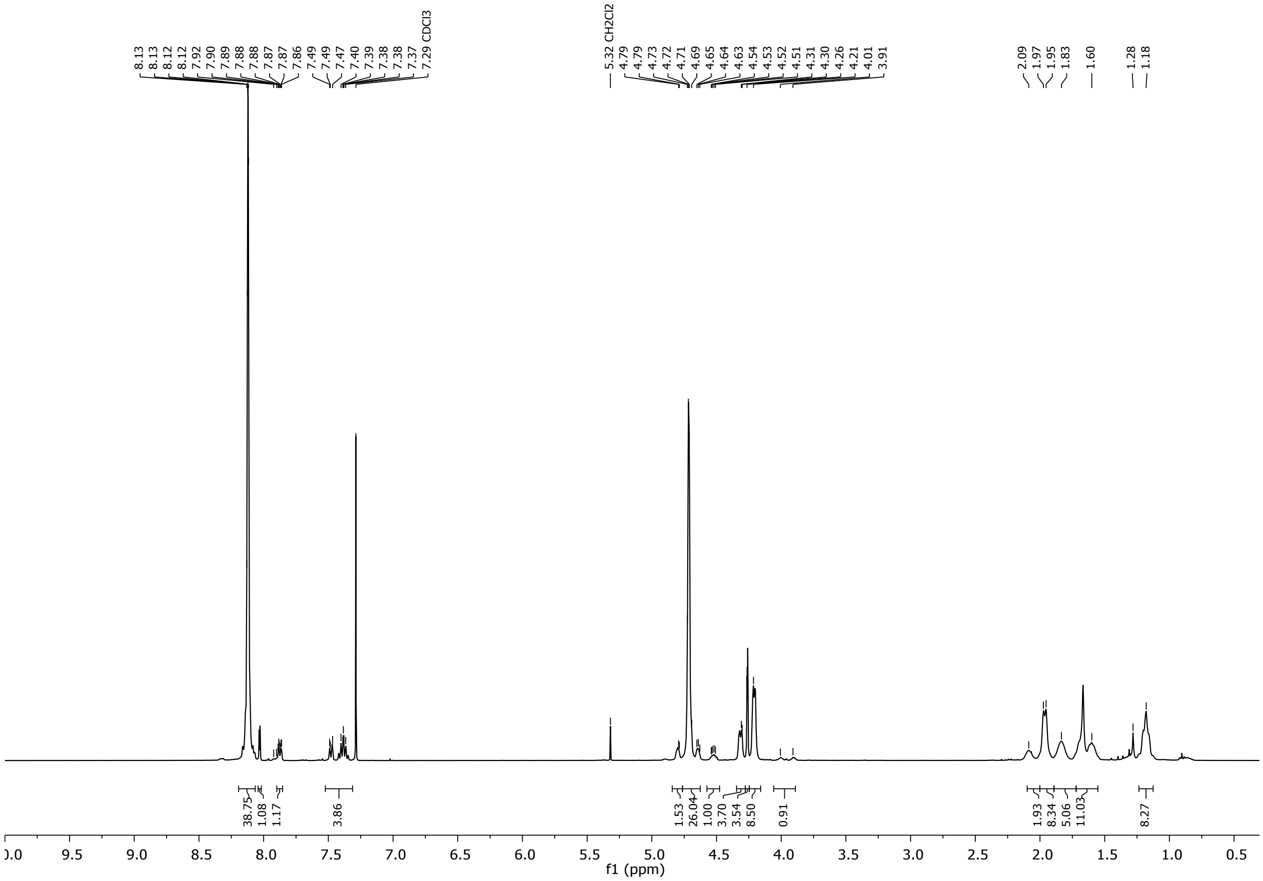

Figure S8. ^1H NMR Spectrum ($CDCl_3$, 400 MHz) of $\mathbf{M_{9k}}$.

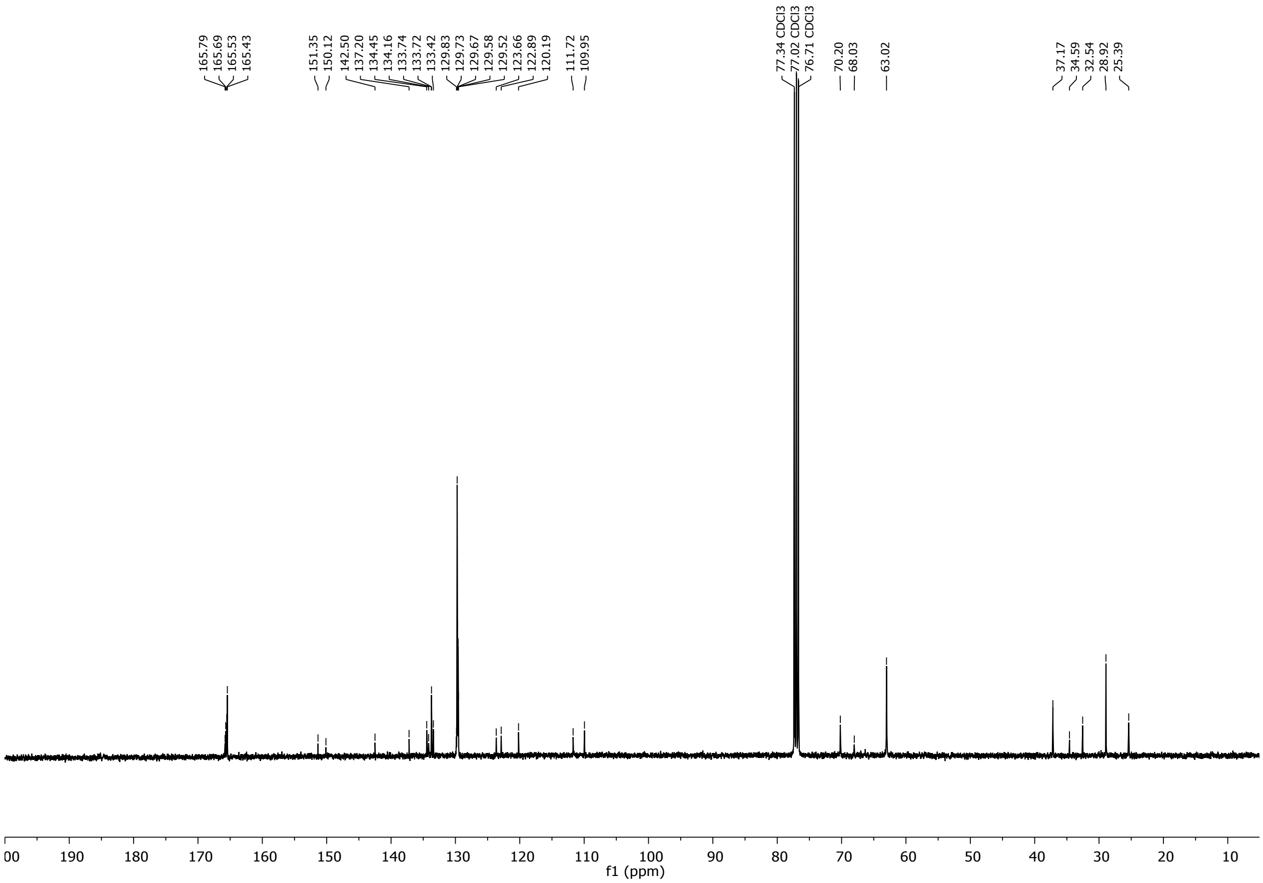

Figure S9. ^{13}C NMR spectrum ($CDCl_3$, 101 MHz) of $\mathbf{M_{9k}}$.

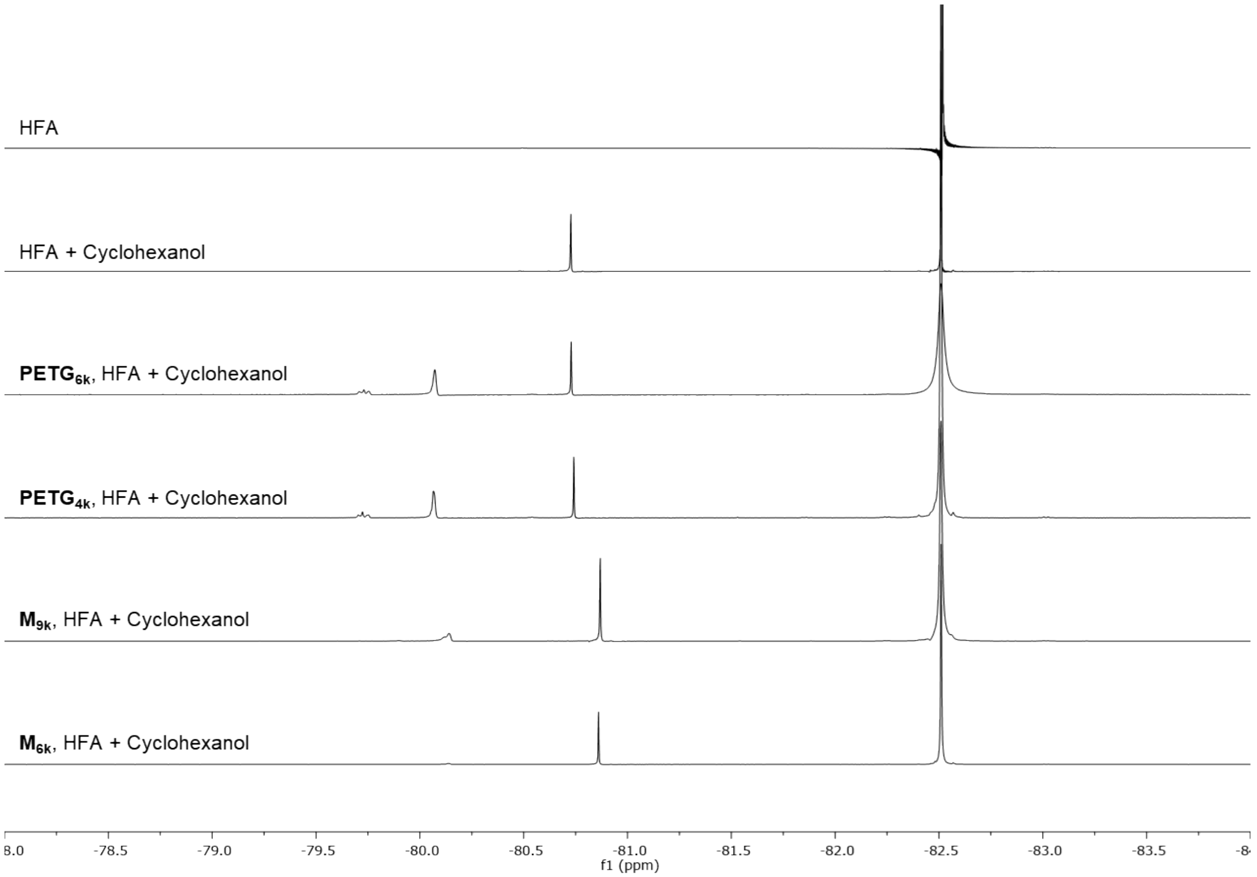

Figure S10. ^{19}F NMR spectra of $\mathbf{PETG_{xk}}$ and $\mathbf{M_{xk}}$ in a 10 wt% hexafluoroacetone (HFA) solution in $CDCl_3$ with the addition of 9.58 M of cyclohexanol as internal standard. The peak at −82.5 ppm corresponds to the HFA hydrate, while the peak at −80.8 ppm indicates the hemiacetal adduct arising from the reaction of cyclohexanol with HFA. The peaks at −80 ppm are the hemiacetal adducts of HFA and the terminal hydroxyl group of $\mathbf{PETG_{xk}}$. The complete disappearance of these peaks in $\mathbf{M_{6k}}$ reveals that this polymer is fully end-capped with Mebip. For $\mathbf{M_{9k}}$, a small fraction (< 20%) of free hydroxyl is still present.

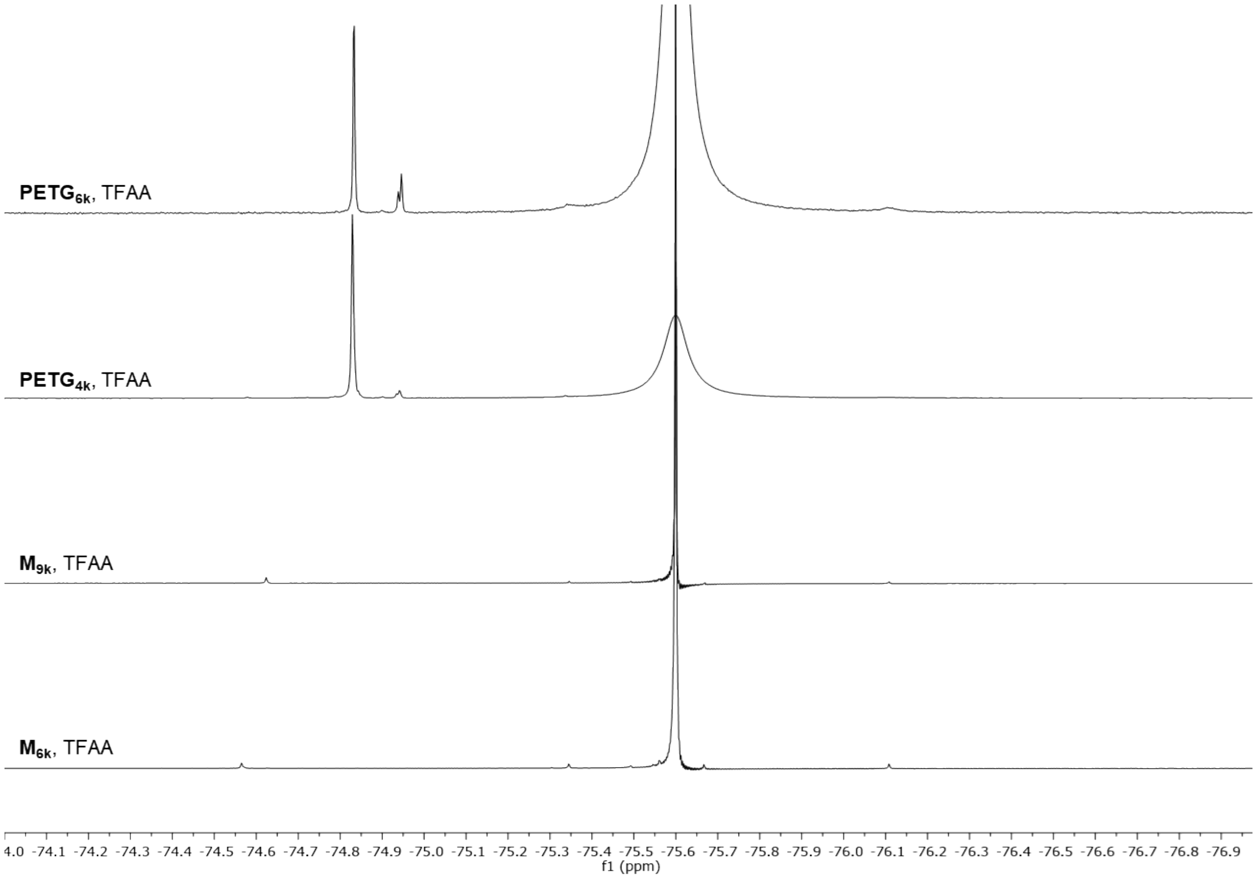

Figure S11. ^{19}F NMR spectra of $\mathbf{PETG_{xk}}$ and $\mathbf{M_{xk}}$ in $CDCl_3$ with the addition of one drop of trifluoroacetic anhydride (TFAA). The peak at −75.6 ppm corresponds to TFAA. The peaks at −74.85 and −74.95 ppm relate to the trifluoroacetyl esters adducts formed by the reaction of terminal alcohols in $\mathbf{PETG_{xk}}$ with TFAA. The disappearance of these peaks in $\mathbf{M_{xk}}$ is a strong indication that the macromonomers are (almost) fully end-capped with Mebip. The small peaks at ca. −74.6 ppm are likely the products formed by minor reactions of a nitrogen of Mebip with TFAA.

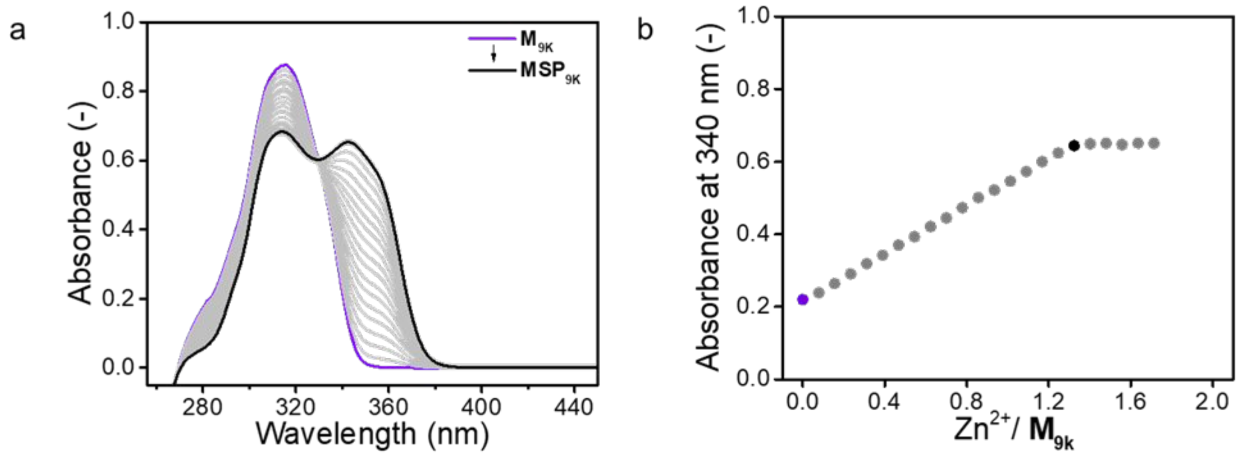

Figure S12. (a) UV-vis absorption spectra recorded during the titration of $\mathbf{M_{9k}}$ using $M_{n,\ NMR}$ = 7492 g mol^{-1} (c = 12.6 µM in $CHCl_3/CH_3CN$, 9:1 v/v; purple trace) with additions of $Zn(OTf)_2$ (c = 654 µM in $CHCl_3/CH_3CN$, 9:1 v/v). The trace changes indicate the formation of the metal-ligand complex $\mathbf{MSP_{9k}}$ (black trace). (b) The plot of the absorbance at 340 nm corresponds to the metal-ligand complex's characteristic band as a function of the Zn^{2+}: $\mathbf{M_{9k}}$ ratio. The data demonstrate that complexation is complete at a Zn^{2+}:$\mathbf{M_{9k}}$ ratio of 1.3:1.

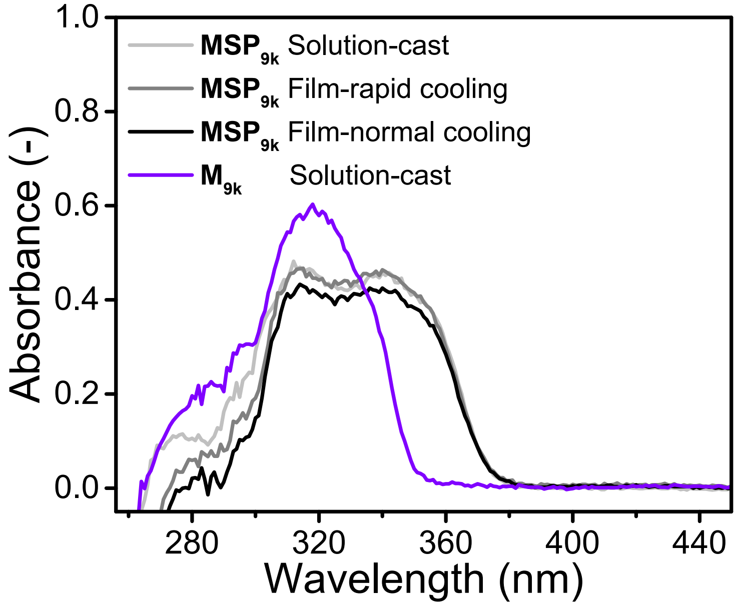

Figure S13. UV-vis absorption spectra of films of $\mathbf{MSP_{9k}}$ produced by spin-coating 100 µL of a 5 mM solution in $CHCl_3/CH_3CN$, 9:1 v/v, $\mathbf{MSP_{9k}}$ solution casting, and $\mathbf{MSP_{9k}}$ thin film with either normal cooling or rapid cooling. Spin-coated samples of PETG and $\mathbf{M_{9k}}$ were prepared analogously. A quartz slide spin-coated with PETG was used as a reference.

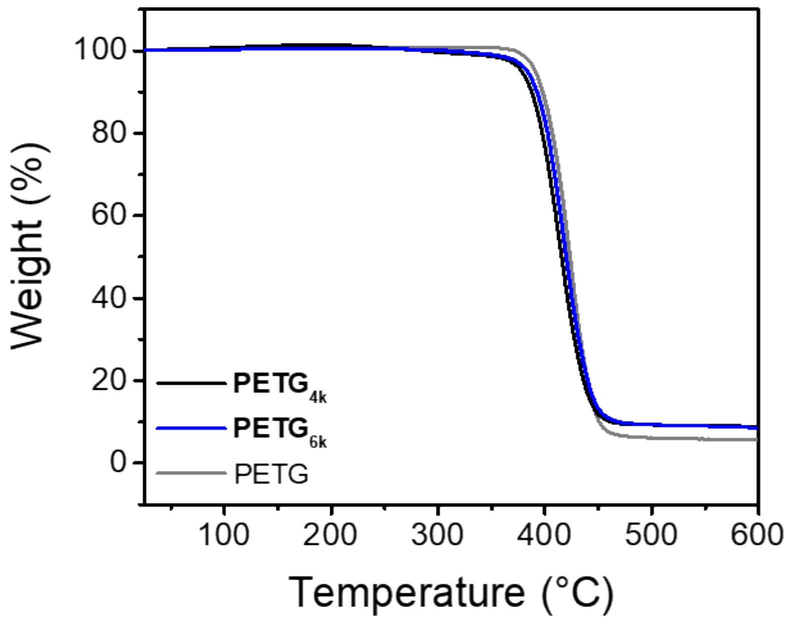

Figure S14. Thermogravimetric analysis (TGA) traces of PETG and the hydroxy-terminated telechelics $\mathbf{PETG_{xk}}$.

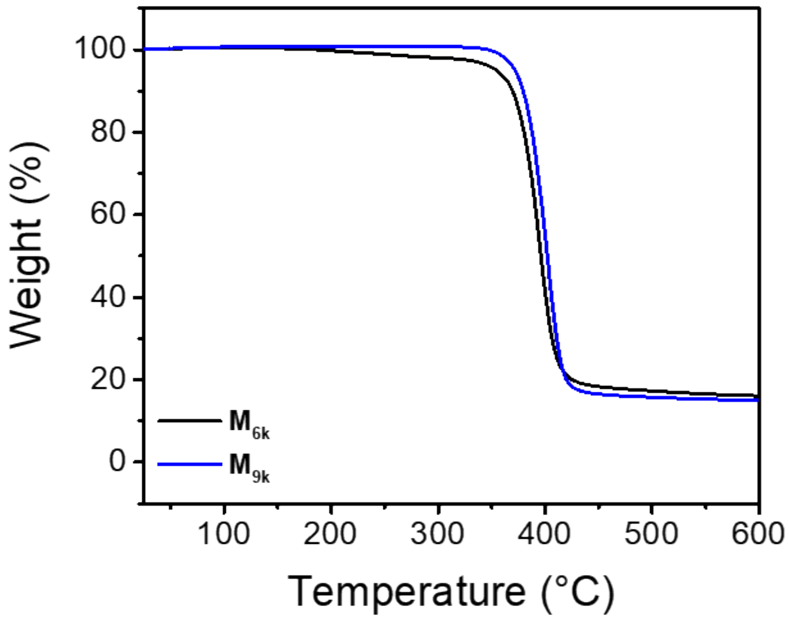

Figure S15. Thermogravimetric analysis (TGA) traces of the macromonomers $\mathbf{M_{xk}}$.

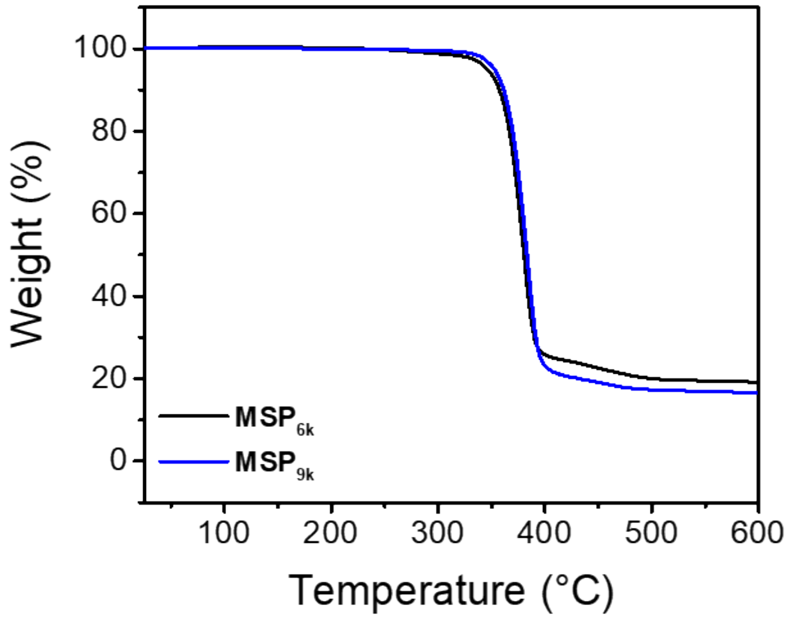

Figure S16. Thermogravimetric analysis (TGA) traces of the metallosupramolecular polymers $\mathbf{MSP_{xk}}$.

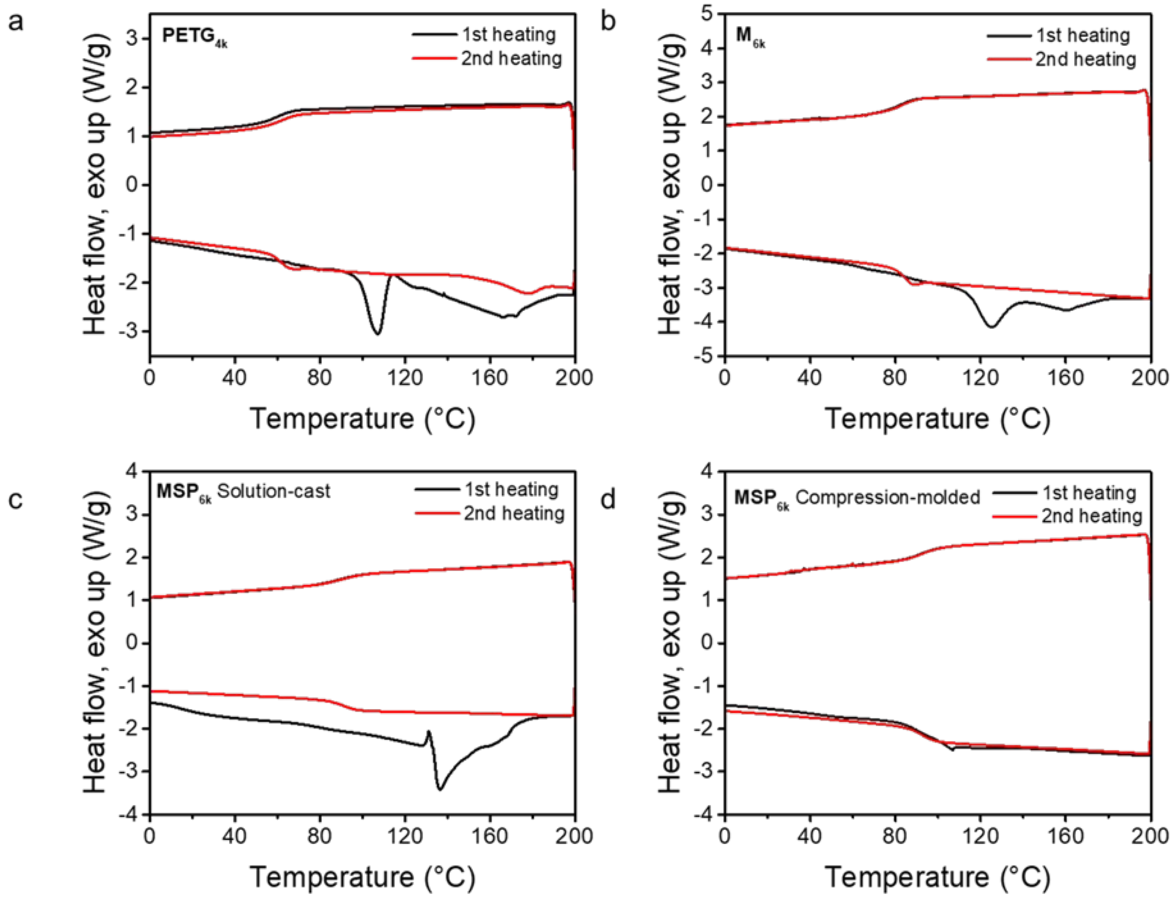

Figure S17. Differential scanning calorimetry (DSC) traces of (a) **PETG$_{4k}$**, (b) **M$_{6k}$**, (c) solution-cast **MSP$_{6k}$**, and (d) compression-molded **MSP$_{6k}$** samples. The first and second heating-cooling cycles are shown. The experiments were conducted with heating and cooling rates of 10 °C min^{-1} under a nitrogen atmosphere.

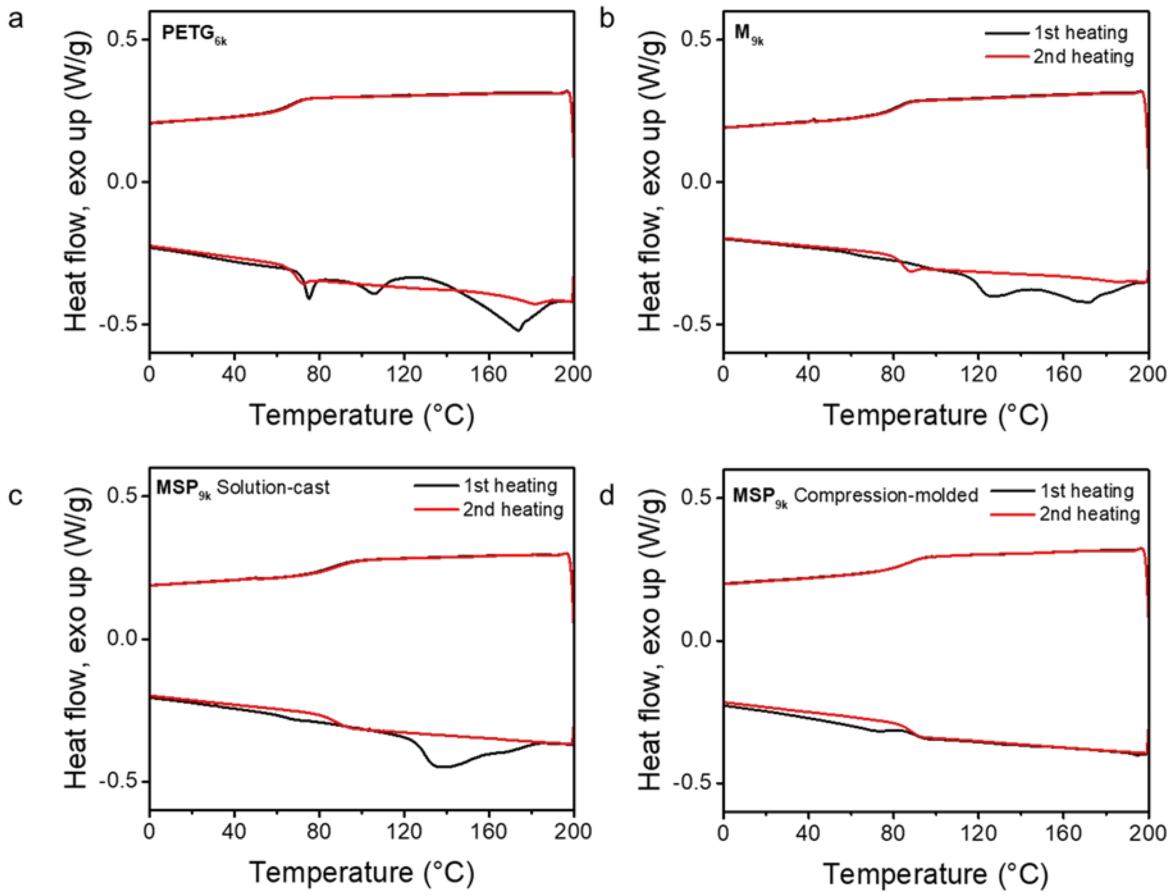

Figure S18. Differential scanning calorimetry (DSC) traces of (a) $\mathbf{PETG_{6k}}$, (b) $\mathbf{M_{9k}}$, (c) solution-cast $\mathbf{MSP_{9k}}$, and (d) compression-molded $\mathbf{MSP_{9k}}$ samples. Shown are the first and the second heating-cooling cycles. The experiments were conducted with heating and cooling rates of 10 °C min^{-1} under a nitrogen atmosphere.

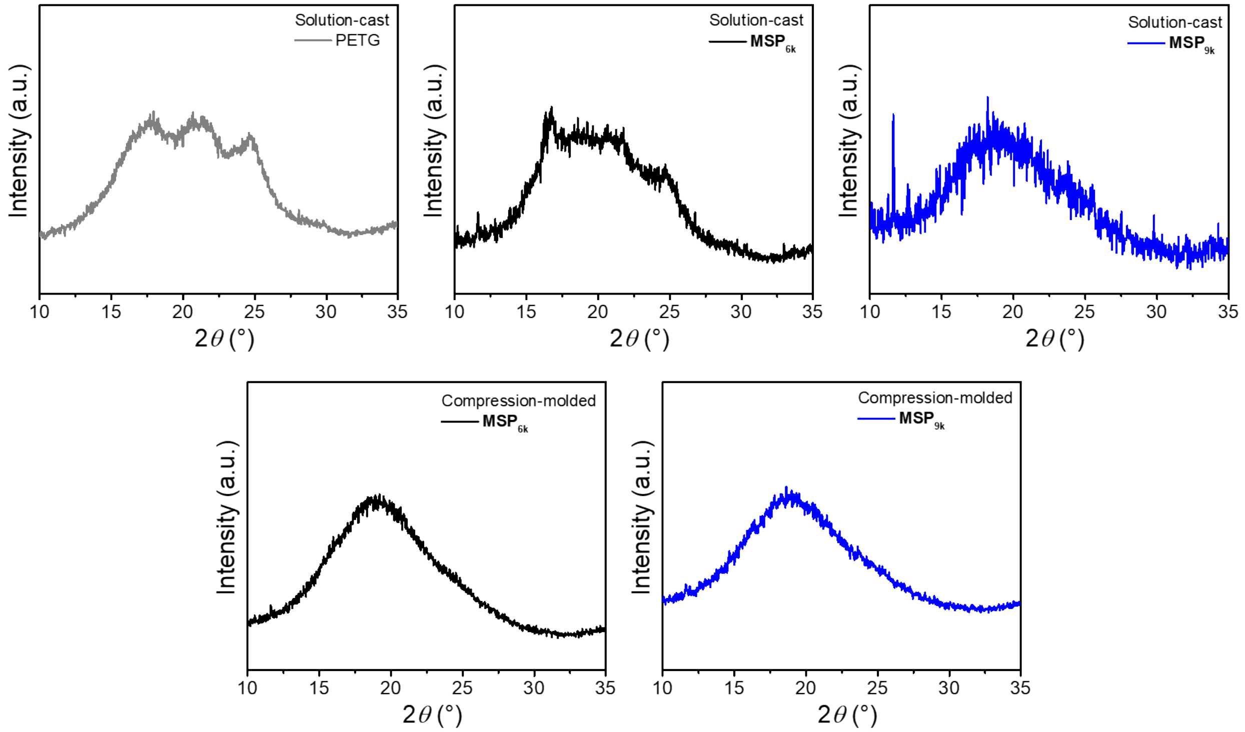

Figure S19. Wide-angle X-ray (WAXS) diffractograms of solution-cast PETG and **MSP$_{\mathbf{xk}}$** films prepared by solution casting and compression molding.

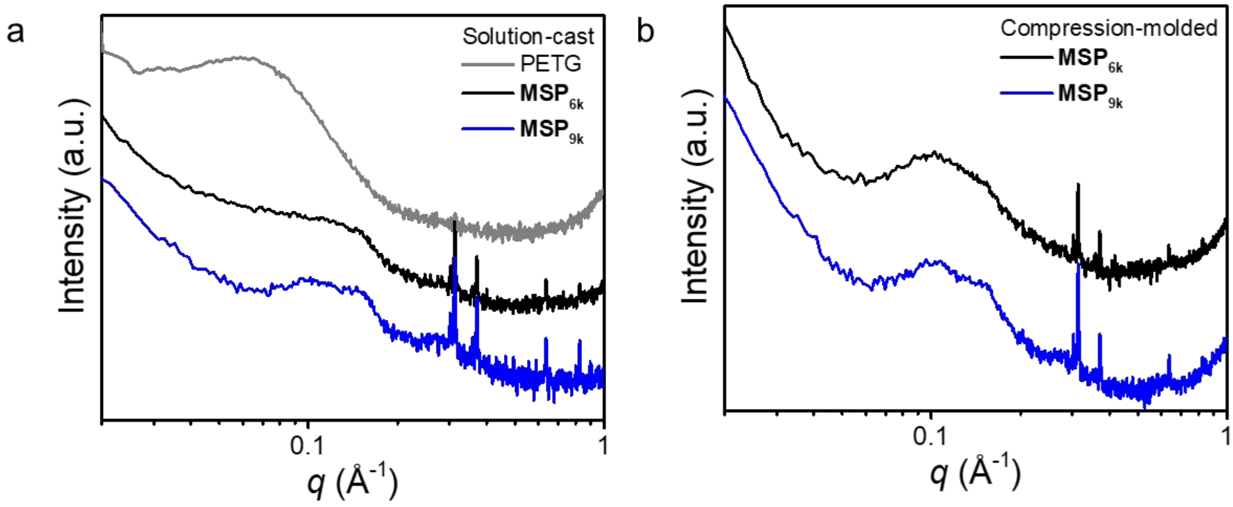

Figure S20. Small-angle X-ray (SAXS) profiles of (a) PETG and **MSP$_{\mathbf{xk}}$** films prepared by solution casting and (b) **MSP$_{\mathbf{xk}}$** films compression molding. Profiles are vertically shifted for clarity. Sharp peaks at q = 0.3 and 0.35 Å^{-1} are present in all samples and are of unknown origin and cannot be ascribed to scattering of the **MSP$_{\mathbf{xk}}$**.

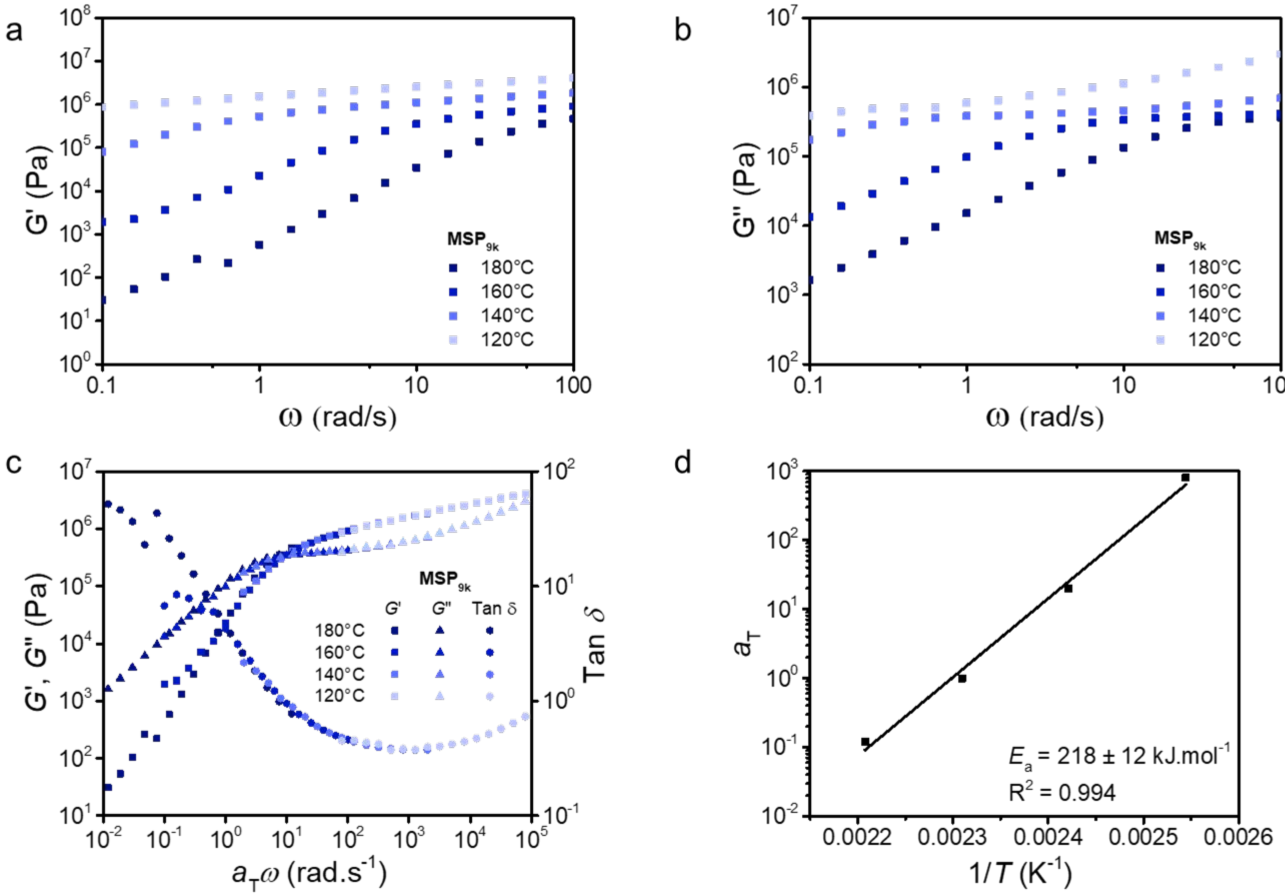

Figure S21. (a) Storage modulus (G') as a function of angular frequency (ω) at 180, 160, 140, and 120 °C. (b) Loss modulus (G'') as a function of angular frequency (ω) at 180, 160, 140, and 120 °C. (c) Master curve after time-temperature superposition (TTS) at the reference temperature of 160 °C. Storage and loss moduli (G' and G'') and loss factor (Tan δ) as a function of shifted angular frequency ($a_T\omega$). (d) Semi-log plot of the horizontal shift factor (a_T) as a function of the inverse of temperature ($1/T$), black squares. The slope of the linear fit (straight line) was multiplied by the universal gas constant (R) to afford an activation energy (E_a) of 218±12 kJ mol^{-1}.

Frequency sweep experiments on **MSP$_{9k}$** were conducted by initially heating the sample to 180 °C to ensure optimal contact with the rheometer plates. After a 10-minute period to achieve thermal homogeneity, the experiment commenced. Subsequent temperatures were attained with a cooling rate of 10 °C min^{-1}, followed by a 10-minute equilibration period to ensure thermal homogeneity. Data were collected across angular frequencies ranging from 100 to 0.1 rad·s^{-1} at a constant strain of γ = 1%.

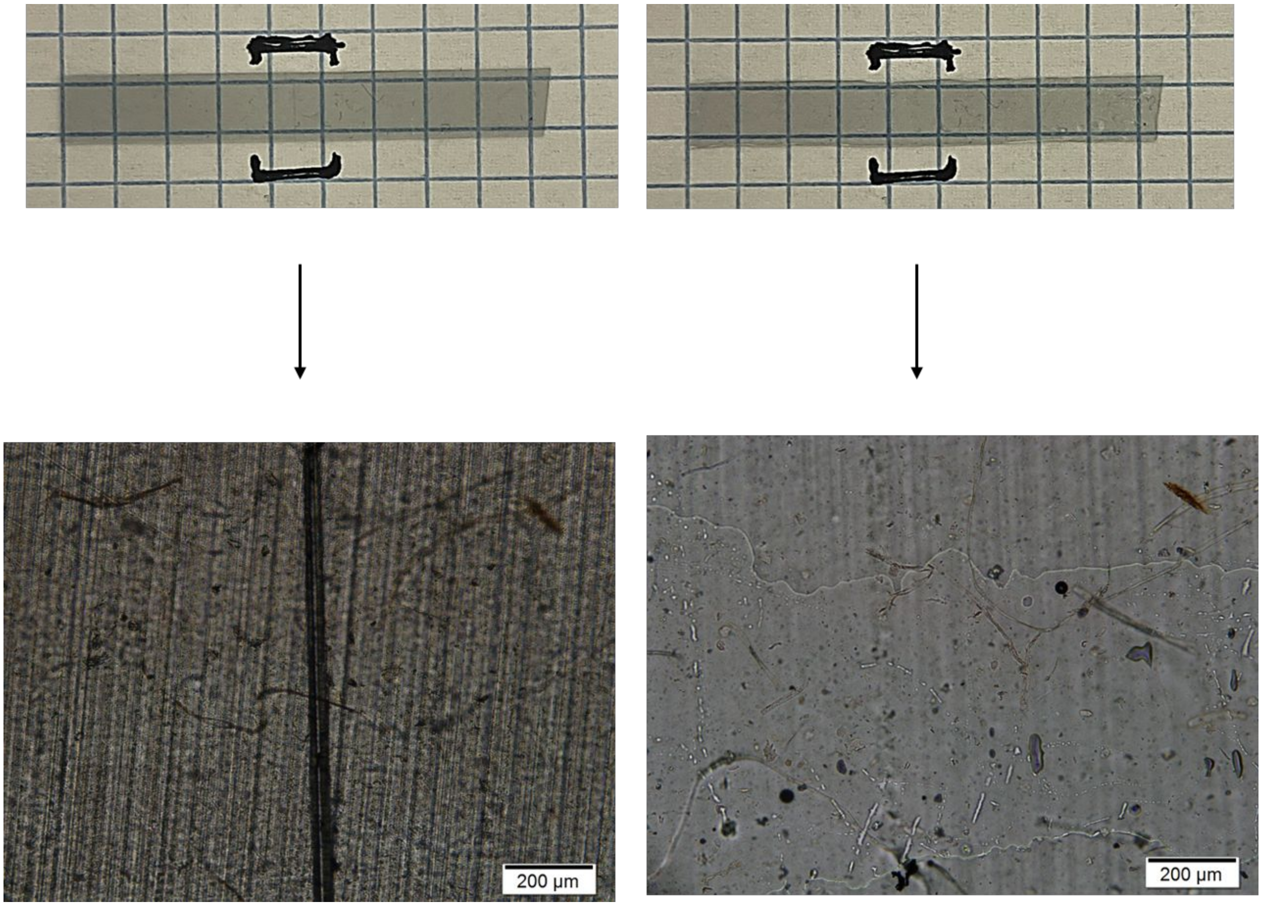

Figure S22. Photographs (top) and optical microscopy images (bottom) of a film of **MSP$_{9k}$** after cutting the sample to ca. 30% of its depth (left) and after heat treatment at 160°C for 2.5 min (right).

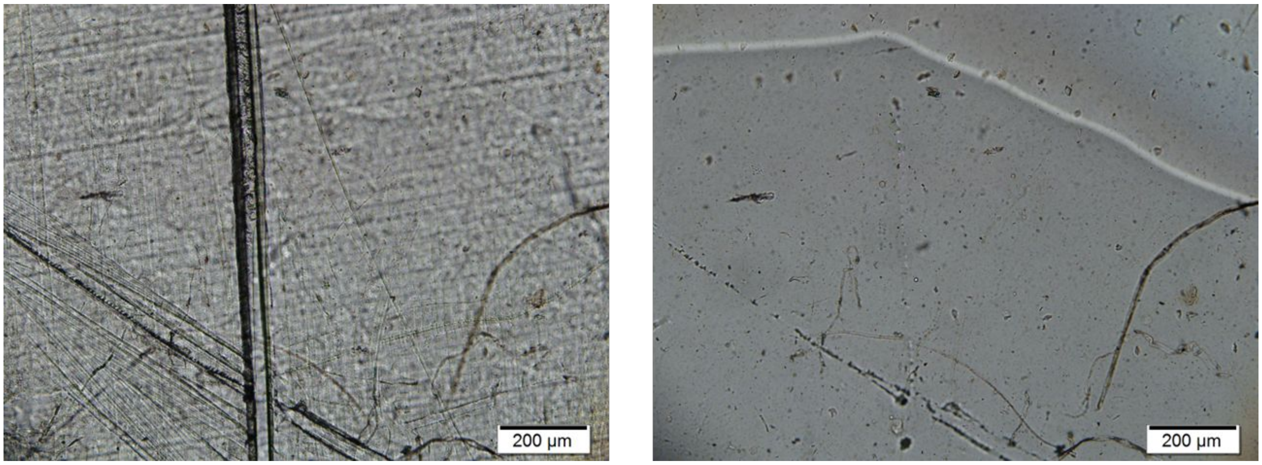

Figure S23. Optical microscopy images of a sample of **MSP$_{6k}$** after scratching (left) and after heat treatment at 160°C for 2 min (right).

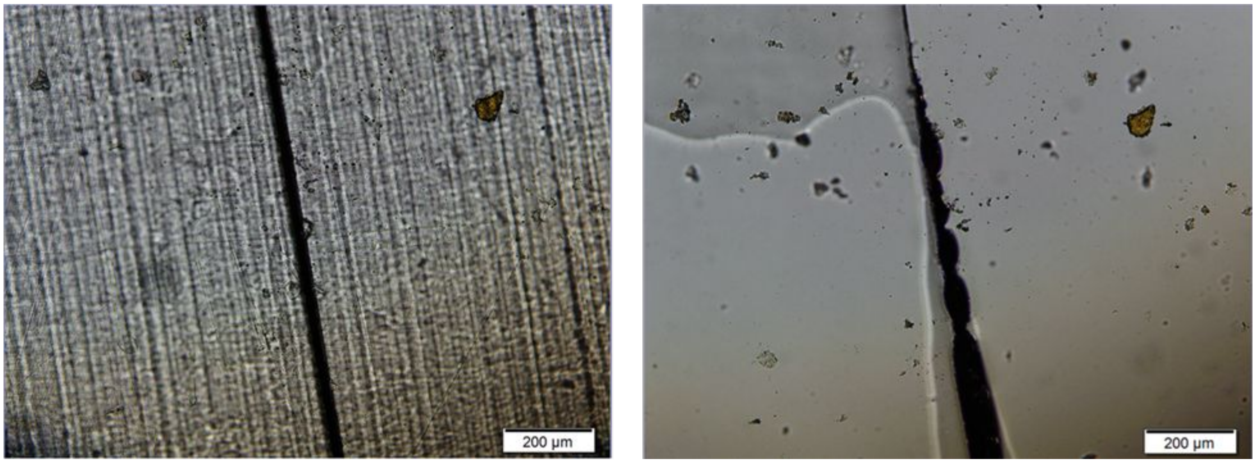

Figure S24. Optical microscopy images of a sample of PETG after scratching (left) and after heat treatment at 160°C for 1 min (right). The film starts to melt before healing takes place.

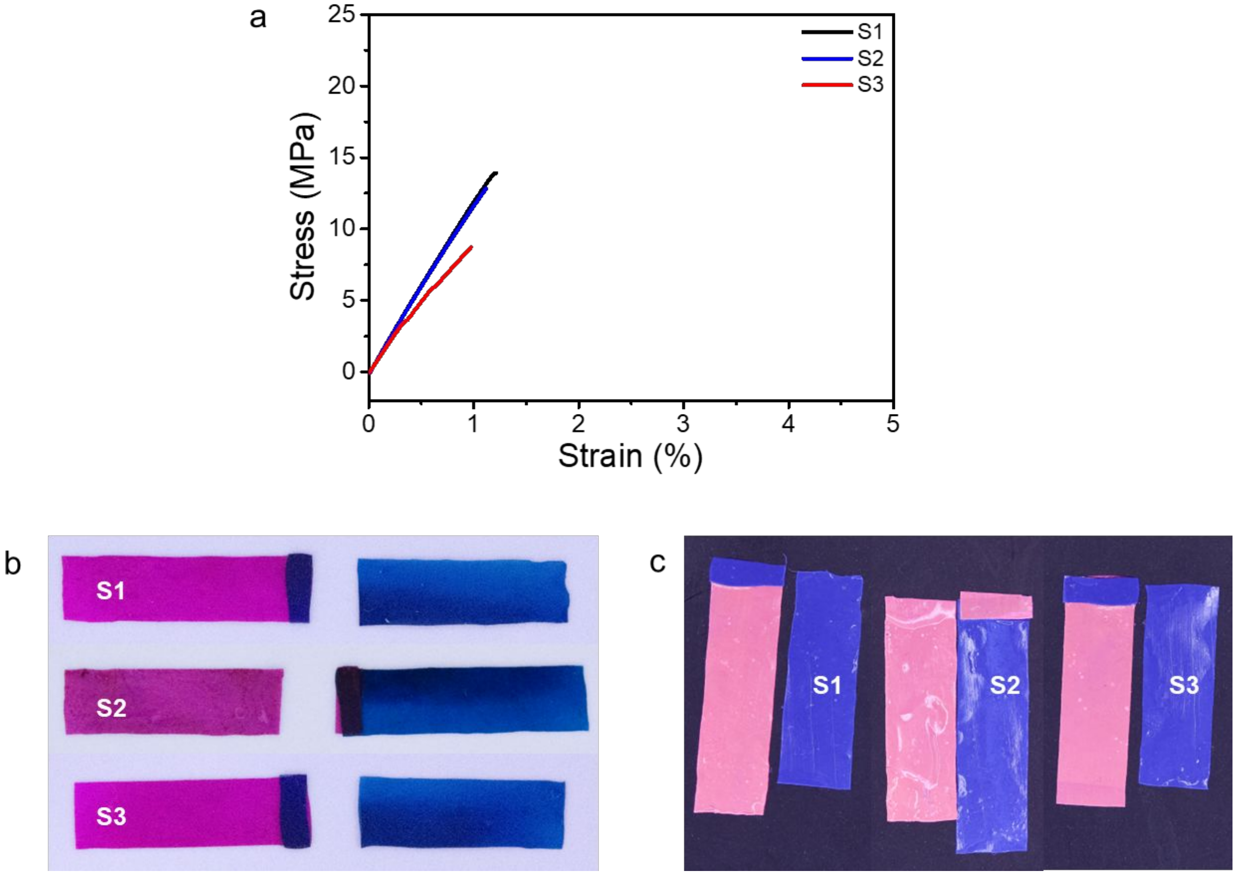

Figure S25. (a) Stress-strain curves of welded samples, recorded at 25 °C. (b) Photograph of samples under normal (c) and UV (d) light after welding and tensile testing. The welded samples are more brittle and less strong than pristine samples; however, all samples demonstrated cohesive failure. The loss in toughness is likely due to inhomogeneities introduced in the sample during the welding process.

Supplementary Tables

Table S1. Molecular weight of the original PETG, the hydroxy-terminated telechelics $\mathbf{PETG_{xk}}$, and the macromonomers $\mathbf{M_{xk}}$.

	$M_{n,NMR}$ (kg mol^{-1})[a]	$M_{n,SEC}$ (kg mol^{-1})[b]	M_w (kg mol^{-1})[b]	Đ (-)[b]
PETG	n.d.[c]	30	48	1.6
$\mathbf{PETG_{6k}}$	3	6	10	1.7
$\mathbf{PETG_{4k}}$	2	4	6	1.5
$\mathbf{M_{9k}}$	7	9	12	1.3
$\mathbf{M_{6k}}$	5	6	8	1.3

[a]Number-average molecular weight calculated by ^{1}H NMR end group analysis.

[b]Determined by size exclusion chromatography (SEC) by using poly(styrene) standards.

[c]n.d = not determined because the peaks of the end groups are not well-resolved in the high molecular weight polymer.

Table S2. Thermal properties of the original PETG, the hydroxy-terminated telechelics $\mathbf{PETG_{xk}}$, the macromonomers $\mathbf{M_{xk}}$, and the $\mathbf{MSP_{xk}}$.

	$T_{g,1st}$[a] (°C)	$T_{m,1st}$[a,b] (°C)	$T_{g,2nd}$[a] (°C)	$T_{m,2nd}$[a,b] (°C)	$T_{d5\%}$[c] (°C)
PETG	83	n.a.	84	n.a.	390
$\mathbf{PETG_{6k}}$	75	105,175	72	180	383
$\mathbf{PETG_{4k}}$	80	108,165	66	180	378
$\mathbf{M_{9k}}$	n.d.	124,172	88	n.a.	371
$\mathbf{M_{6k}}$	n.d.	125,160	89	n.a.	354
$\mathbf{MSP_{9k}}$ solution-cast	n.d.	148	92	n.a.	350
$\mathbf{MSP_{9k}}$ compression-molded	90	n.a.	90	n.a.	352
$\mathbf{MSP_{6k}}$ solution cast	n.d.	145	93	n.a.	335
$\mathbf{MSP_{6k}}$ compression-molded	95	n.a.	95	n.a.	346

[a]The glass transition (T_g) and melting (T_m) temperatures were determined by differential scanning calorimetry (DSC) (shown are results for the 1st and 2nd heating).

[b]Temperatures quoted indicate the maximum of the melting transition; n.a. = not applicable.

[c]The temperature at which a weight loss of 5 wt% ($T_{d5\%}$) was measured by thermogravimetric analysis (TGA). The measurements were performed with a heating rate of 10 °C min^{-1} under a N_2 atmosphere.

Table S3. Thermal and mechanical properties of the original PETG and the **MSP_{xk}**.

	T_g (°C)[a]	E’ at 25°C (MPa)[a]	Failure temp. (°C)[a]	Young’s Modulus E (MPa)[b]	Yield stress σ_y (MPa)[b]	Stress at break σ_b (MPa)[b]	Yield point ε_y (MPa)[b]	Elongation at break ε_b (%)[b]	Toughness (kJ m^{-3})[b]
PETG	86±2	1053±91	111±2	1400±43	36±4	33±4	4±0.3	259±51	6602±2351
**MSP_{6k}**	103±0.5	1461±161	155±0.2	1240±43	n.a[c]	18±5	n.a[c]	1.6±0.4	13±3
**MSP_{9k}**	99±3	1224±171	156±7	1061±146	n.a[c]	31±3.4	n.a[c]	3.3±0.5	54±14

All data represent averages of n = 3 individual measurements ± standard deviation.

[a]Measured by DMA.

[b]Measured by stress-strain experiments at 25 °C with a strain rate of 150% min^{-1}.

[c]n.a. = not applicable.

Table S4. Mechanical data of the healing experiments carried out with **MSP_{9k}**.

	Young's Modulus (MPa)	Tensile strength (MPa)	Strain at break (%)	Toughness ($kJ \cdot m^{-3}$)	Healing efficiency (%)[a]
Original	1061±146	31±3.4	3.3±0.5	54±14	n.a.
Damaged[b]	1100±41	18±3.0	1.7±0.3	15±5	n.a.
Healed[c]	1124±145	29±0.9	2.8±0.3	44±6	94±11

Data represents averages of n = 3 individual measurements ± standard deviation.

[a]Healing efficiency was calculated by dividing the stress at break of the healed by the original sample. The error was calculated by error propagation.

[b]Damaged samples were scratched to a depth of around 30 % of the original sample thickness using a razor blade attached to a caliper for precise depth control.

[c]Healed samples were exposed to heat at 160°C for around 2.5 min until the scratch disappeared.

Table S5. Mechanical data of the welded samples.

	Young's Modulus (MPa)	Tensile strength (MPa)	Strain at break (%)
S1	1264	14	1.2
S2	1258	13	1.1
S3	1095	9	1.0

Supporting References

(1) Beck, J. B.; Ineman, J. M.; Rowan, S. J. Metal/ligand-induced formation of metallo-supramolecular polymers. *Macromolecules* **2005**, *38* (12), 5060-5068.

(2) Burnworth, M.; Tang, L.; Kumpfer, J. R.; Duncan, A. J.; Beyer, F. L.; Fiore, G. L.; Rowan, S. J.; Weder, C. Optically healable supramolecular polymers. *Nature* **2011**, *472* (7343), 334-337.

(3) Fulmer, G. R.; Miller, A. J. M.; Sherden, N. H.; Gottlieb, H. E.; Nudelman, A.; Stoltz, B. M.; Bercaw, J. E.; Goldberg, K. I. NMR Chemical Shifts of Trace Impurities: Common Laboratory Solvents, Organics, and Gases in Deuterated Solvents Relevant to the Organometallic Chemist. *Organometallics* **2010**, *29* (9), 2176-2179.

(4) Keizer, H. M.; van Kessel, R.; Sijbesma, R. P.; Meijer, E. W. Scale-up of the synthesis of ureidopyrimidinone functionalized telechelic poly(ethylenebutylene). *Polymer* **2003**, *44* (19), 5505-5511.

(5) Kumpfer, J. R.; Wie, J. J.; Swanson, J. P.; Beyer, F. L.; Mackay, M. E.; Rowan, S. J. Influence of Metal Ion and Polymer Core on the Melt Rheology of Metallosupramolecular Films. *Macromolecules* **2012**, *45* (1), 473-480.
